# Fine‐Grained Concreteness Effects on Word Processing and Representation Across Three Tasks: An ERP Study

**DOI:** 10.1111/psyp.70074

**Published:** 2025-05-23

**Authors:** Maria Montefinese, Antonino Visalli, Alessandro Angrilli, Ettore Ambrosini

**Affiliations:** ^1^ Department of Developmental Psychology and Socialisation University of Padova Padova Italy; ^2^ IRCCS San Camillo Hospital Venice Italy; ^3^ Department of Biomedical, Metabolic and Neuroscience University of Modena and Reggio Emilia Reggio Emilia Italy; ^4^ Department of General Psychology University of Padova Padova Italy; ^5^ Padova Neuroscience Center, University of Padova Padova Italy; ^6^ Department of Neuroscience University of Padova Padova Italy

**Keywords:** concreteness, event‐related potentials, semantic representation, task demands, word

## Abstract

People process concrete words more quickly and accurately than abstract ones—the so‐called “concreteness effect.” This advantage also reflects differences in how the brain processes and stores concrete versus abstract words. In this electrophysiological study, we treated word concreteness as a continuous variable and examined its effects on ERPs across three tasks with distinct processing demands (semantic, affective, grammatical). Behavioral results revealed task‐dependent concreteness effects: in the semantic task, reaction times were faster for words at both concreteness extremes, and the classical linear advantage emerged for concrete words. Mass univariate ERP analyses revealed distinct spatiotemporal patterns of task‐dependent concreteness effects. In the semantic task, we identified three significant clusters reflecting increased parietal N2/P3‐like and sustained bilateral fronto‐temporal negativity ERPs and decreased central N400‐like ERP for abstract words. By contrast, the affective task elicited an increased parietal P600‐like ERP for abstract words. Moreover, results from multivariate representational similarity analysis and an intersection analysis revealed that concreteness is encoded in ERP spatiotemporal patterns from 450 ms onwards, regardless of task, suggesting its role not only as an organizational principle in semantic representation, but also as a factor influencing downstream word processing and univariate ERP concreteness effects. Our findings challenge and extend existing theories like the dual coding and context availability ones, highlighting the importance of treating concreteness as a continuous variable and considering task context in word processing studies. This approach, enabled by advanced analytical techniques, provides a more nuanced understanding of how the brain processes and represents words.

## Abstract 2

This study reveals that our brains process words differently based on how concrete or abstract they are, and this effect changes depending on the task at hand. By using advanced brain imaging techniques and treating word concreteness as a spectrum rather than a simple yes‐or‐no category, we uncovered new insights into how our brains represent and process words based on their concreteness. These findings challenge existing theories about word processing and highlight the importance of considering both the nature of words and the context in which they are used.

## 1 Introduction

Every day we communicate about entities of the world that can be concrete, such as *pizza*, but also more abstract, such as *justice*, which represent almost 70% of the words we use daily (Bechtold et al. 2023 ). The dimension that determines whether an entity is concrete or abstract has been termed *concreteness*. Concreteness indicates the degree to which a word refers to an entity that can be perceived directly through the senses (i.e., vision, hearing, touch, smell, and taste) (Brysbaert et al. 2014). This property is an organizing principle of semantic representation—the memory store of our knowledge (Montefinese 2019; Reilly et al. 2024). This dimension is usually derived by simply asking participants to rate the concreteness of a word on a Likert scale (Brysbaert et al. 2014; Fairfield et al. 2017). Low scores indicate highly abstract words referring to entities that lack clearly perceivable referents, are more strongly reliant on interoception (i.e., sensations inside the body) (Connell et al. 2018; Montefinese et al. 2020) and have more emotional content and less imageability. By contrast, high scores on the concreteness scale indicate highly concrete words referring to single, bounded, identifiable entities perceived through the senses (Borghi et al. 2017). Indeed, concrete concepts are acquired earlier and mostly through direct experience with the world, whereas abstract concepts are acquired later and mostly through language and social interaction (see recent review by Dove et al. 2022).

Behavioral research over several decades has found that concrete words are processed more quickly and accurately than abstract words (see review by Huang and Federmeier 2015). This advantage is labeled the concreteness effect and has been observed in a variety of tasks. For example, as compared to abstract ones, concrete words are associated with faster response times (RT) in lexical decision tasks (Bleasdale 1987; Kroll and Merves 1986; but see also Kousta et al. 2011), are easier to encode and retrieve (Miller and Roodenrys 2009; Romani et al. 2008), are easier to make associations with (de Groot 1989), and are more thoroughly described in definition tasks (Sadoski et al. 1997). However, previous studies have shown that when psycholinguistic variables like word frequency, age of acquisition, familiarity, and emotional valence are not properly controlled, they can obscure or even reverse the classic concreteness effect (Kousta et al. 2011). For example, abstract words tend to be more emotionally valenced than concrete words. When this emotional richness is not accounted for, abstract words can actually be processed faster, masking the typical concreteness advantage. Similarly, surface properties of words like their orthographic form and neighborhood density can influence processing times (Mkrtychian et al. 2021). If concrete and abstract words differ systematically in these surface features and they are not matched across conditions, any observed differences could be due to these lexical characteristics rather than concreteness per se.

Two prominent theories have emerged to elucidate the concreteness effect. The *dual‐coding account*, put forth by Paivio (1990), posits the existence of two processing systems: a verbal symbolic system responsible for handling linguistic information and an imagery system for nonverbal content. For concrete concepts with tangible referents that can be directly experienced, the theory proposes both the verbal and imagery codes are activated, allowing for dual coding. Conversely, for abstract concepts lacking physical instantiations, only the verbal code is engaged since these concepts lack perceptual information to populate the imagery code. This asymmetry, with concrete words utilizing both codes but abstract words relying solely on the verbal code, is a key tenet of the theory used to account for the well‐established processing advantages observed for concrete compared to abstract concepts. By contrast, the *context‐availability model* (Schwanenflugel et al. 1992) posits that comprehension is dependent on context, which can be derived from either the preceding discourse or the individual's semantic knowledge. Concrete words are thought to have stronger and more extensive connections to this contextual knowledge within semantic representations compared to abstract words (Bechtold et al. 2023). Consequently, the worst performance for abstract words is attributed to the relative scarcity of associated contextual information in their semantic representation (Schwanenflugel et al. 1992). Indeed, abstract concepts may be linked to a broader range of situations than concrete ones (Galbraith and Underwood 1973), potentially due to their greater number of possible meanings (i.e., polysemy or *semantic diversity*) compared to concrete words (Hoffman et al. 2013). This wider associative network can lead to increased interference between competing situations, making it more challenging to retrieve a specific context for an abstract word compared to a concrete one. It is important to note that in real‐world scenarios, unlike in laboratory settings where words are often presented in isolation, abstract words are typically processed within an existing relevant context. This contextual embedding could potentially facilitate the processing of abstract words by mitigating the interference between competing situations. Indeed, some research has shown that the performance gap between concrete and abstract words narrows when abstract words are provided with sufficient contextual support (Bechtold et al. 2023; Holcomb et al. 1999; Schwanenflugel 1992).

The ontological distinction between abstract and concrete words is also reflected in their different processing and representation in the human brain. Indeed, many functional neuroimaging studies show that the left and right cerebral hemispheres process concrete and abstract words differently (Montefinese, Pinti, et al. 2021; Pexman et al. 2007; Sabsevitz et al. 2005). Researchers generally agree that two brain systems are important for processing different types of words: Abstract words seem to rely more on the verbal‐language system, while concrete words seem to rely more on the imagery and perceptual systems (Wang et al. 2010). These results suggest that abstract and concrete words might be represented differently in the brain, reflecting varying degrees of similarity in their meanings (Montefinese 2019). To gain deeper insights into these representational patterns, behavioral and neuroimaging studies have used representational similarity analysis^1^ (Kriegeskorte et al. 2008a), an analytical framework that allows for investigating how abstract and concrete words are represented in the brain and, therefore, how these representations might influence the observed neural responses and behavioral effects. These studies have explored various dimensions of similarity –such as semantic, perceptual, and contextual features—in the organization of abstract and concrete word representations. Still, these studies have not provided unequivocal evidence in support of a single dominant dimension governing the semantic organization and brain representation of abstract and concrete words (Crutch and Warrington 2005; Meersmans et al. 2020; Montefinese et al. 2018, 2024; Montefinese, Pinti, et al. 2021).

Electroencephalography (EEG), with a special focus on the event‐related potential (ERP) technique, stands as an invaluable tool for investigating the nuanced distinctions in how abstract and concrete words are processed, largely owing to its exceptional temporal precision. In contrast to conventional reaction time measures, ERP studies offer a window into the intricate time course of various cognitive processes that collectively shape behavioral responses (Huang and Federmeier 2015). The body of ERP research on the concreteness effect exhibits remarkable consistency across a diverse array of cognitive tasks. These include imageability ratings, lexical decisions, go‐no go tasks, congruence and semantic judgments, and implicit and explicit memory tasks (Adorni and Proverbio 2012; Dufau et al. 2015; Kanske and Kotz 2007; Kellenbach et al. 2000; Lee and Federmeier 2008; Swaab et al. 2002; West and Holcomb 2000; Xiao et al. 2012).

Overall, the ERP studies consistently unveil an N400 component, characterized by a negative waveform observed predominantly at centroparietal electrode sites and peaking around 400 ms post‐stimulus. This N400 component is intricately linked with the processing of meaning, particularly in terms of semantic access (for an extensive review, see Kutas and Federmeier 2011). Notably, investigations into the concreteness effect within ERP studies reveal a larger N400‐like component elicited by concrete words compared to the abstract ones (e.g., West and Holcomb 2000; Welcome et al. 2011; Xiao et al. 2012). This effect typically manifests within a time window spanning from 300 to 500 ms after the onset of the presentation of the target stimulus. One plausible interpretation aligns with the context availability theory, suggesting that concrete words elicit larger N400 amplitudes due to the post‐lexical processing to integrate the denser context‐related associative networks of concrete words. An alternative explanation proposes that the larger N400 component for concrete words (with a more frontal distribution) reflects stronger semantic activation due to the integration of the multimodal features characterizing the richer semantic networks of concrete words (Bechtold et al. 2022; Barber et al. 2013). Importantly, the N400 difference between abstract and concrete words can be diminished when these words are presented within a predictive context (Bechtold et al. 2022; Holcomb et al. 1999). This reduction in the concreteness effect is predicted by both the theoretical explanations just described above, as it may be explained by a facilitation in the semantic integration processes that occur when semantic information is pre‐activated by the context (Matsumoto et al. 2005; Lau et al. 2008).

Additionally, a concreteness effect emerges in the form of a sustained N700, starting around 500 ms and lasting up to 1000 ms post‐stimulus. When this N700 shows a heightened amplitude, it typically signifies the top–down retrieval of information (Adorni and Proverbio 2012) or, in situations where the task demands it, the activation of mental imagery processes (Gullick et al. 2013). This N700 concreteness aligns with the dual‐coding theory and likely mirrors strategic retrieval of visual information occurring at a later stage in the semantic processing. This phenomenon tends to be more pronounced when dealing with concrete words due to their greater imageability and when tasks specifically require the engagement of imagery processes (Barber et al. 2013; Bechtold et al. 2023; West and Holcomb 2000).

Nevertheless, it is important to acknowledge that specific findings in ERP studies can exhibit variability. Some researchers have suggested that this inconsistency could be attributed to several confounding variables (e.g., word frequency, age of acquisition) and the nature of the tasks involved, which may affect the concreteness effect on ERP responses (Huang and Federmeier 2015; Pexman et al. 2007). This is particularly crucial given that semantic representation is a dynamic and flexible construct, continually shaped by the demands of the context and task. It encompasses a wide range of information, spanning perceptual, motor, affective, situational, and linguistic dimensions to varying degrees (Connell 2019). Regrettably, only a limited number of ERP studies have delved into this issue, revealing that the presence and magnitude of the concreteness effect might be sensitive to the type of task or depth of encoding employed (Gullick et al. 2013; Welcome et al. 2011; West and Holcomb 2000; Xiao et al. 2012), as also pointed out by a recent review by Federmeier (2022). Indeed, it tends to be more pronounced in tasks that emphasize mental imagery and the explicit processing of semantic properties, as opposed to tasks that prioritize the surface level characteristics of words.

Another pivotal aspect of the concreteness effect lies in its non‐dichotomous nature; concrete and abstract words, while distinct, do not neatly fall into separate categories, and there exists no clear‐cut boundary delineating them. Instead, we should envision word concreteness as a continuous abstract‐concrete spectrum, approximated by averaging participants' ratings (Borghi et al. 2017). The ERP studies mentioned above thus far have predominantly handled concreteness in a categorical manner, potentially oversimplifying its nuanced and variable nature. Therefore, to gain a deeper insight into the temporal dynamics of the concreteness effect in word processing, it becomes imperative to explore the continuous influence of this variable on both behavioral and ERP responses.

To our knowledge, only two studies (Dufau et al. 2015; Winsler et al. 2018) have ventured into examining the continuous impact of concreteness, employing distinct analytical approaches (item‐level partial regression analysis, Dufau et al. 2015; linear mixed effect models, Winsler et al. 2018). Dufau et al. (2015) conducted a go/no go lexical decision task with visually presented words, revealing an N400 concreteness effect akin to earlier ERP studies. They observed larger late negativities for more concrete words within the 300–500 ms window. In contrast, Winsler et al. (2018) diversified the types of tasks, employing both go/no go lexical decision and semantic categorization tasks with auditory words. Across these tasks, they uncovered robust concreteness effects occurring between 400 and 900 ms after post‐word onset, with a widespread distribution over central scalp sites. Intriguingly, this effect was more pronounced during semantic categorization compared to lexical decision tasks between 400 and 800 ms, supporting the idea that explicit processing of concreteness might elicit more pronounced ERP responses. While the latter study represents a valuable advancement in understanding ERP effects, the use of auditory word presentation might constrain the ability to unveil the intricate temporal dynamics underlying the processing of abstract and concrete words across various tasks. Hence, a comprehensive investigation into these dynamics, especially within the realm of visually presented words, remains a critical pursuit.

The complexity of the concreteness effect, underlined above, thus underscores the need for a comprehensive investigation into the multifaceted nature of word processing, taking into account the intricate interplay of various influential factors. Such an approach promises to yield a deeper understanding of the concreteness effects and its implications for cognitive neuroscience. In the current study we investigated the subtle nuances of the concreteness effect by examining participants' behavioral and ERP responses in a fine‐grained manner. To achieve this, we manipulated concreteness as a continuous measure and employed three distinct tasks, each designed to activate different facets of word information (grammatical, affective, and semantic, respectively). In the current study we investigated the subtle nuances of the concreteness effect by examining participants' behavioral and ERP responses in a fine‐grained manner. To achieve this, we manipulated concreteness as a continuous measure and employed three distinct tasks, each designed to activate different facets of word information (grammatical, affective, and semantic, respectively). This approach allowed us to manipulate the depth of encoding and access to information in semantic representation. Specifically, at one end of the spectrum, the semantic task required explicit access to concreteness information within the semantic representation, as participants had to judge whether each word denoted a concrete or abstract concept. On the opposite end, we employed a grammatical task, in which participants were asked to indicate the grammatical gender of words. This task deliberately made word concreteness a task‐irrelevant dimension of words, ensuring that its processing was not useful for solving the task (Montefinese, Ambrosini, et al. 2019). Finally, the affective task ensured access to semantic representations by requiring participants to judge the emotional valence (positive or negative) of each word. While this task did not explicitly probe concreteness, emotional associations are considered an embodied aspect of semantic representations, especially for abstract concepts which tend to be more emotionally valenced (Connell et al. 2018; Montefinese et al. 2020). Therefore, the affective task granted access to semantic information, including potential differences in emotional embodiment between concrete and abstract words. However, unlike the semantic task, the processing of concreteness itself remained implicit and not relevant for performing the affective task, as participants were not explicitly evaluating this dimension. By employing these three tasks varying in their semantic processing demands, we could systematically investigate how the behavioral and neural signatures of the concreteness effect are modulated by the depth at which semantic information, particularly concreteness, is accessed and encoded.

At the behavioral level, we anticipated a quadratic concreteness effect on reaction times when an explicit processing of word concreteness is required (i.e., in the semantic task). Indeed, according to Piéron's Law (Pins and Bonnet 1996), semantic decisions should be easier at the extreme values of the concreteness dimension (i.e., faster RT for highly abstract and highly concrete words) and harder at intermediate values (i.e., slower RT for words with moderate concreteness). We also examined the linear effect of concreteness to test the classical advantage in the processing of concrete words compared to the abstract ones, for which we expect faster RT for more concrete words compared to the more abstract ones, when processing the concreteness is task‐relevant after controlling for confounding variables.

As regards the ERP data, in accordance with both the dual‐coding theory (Paivio 1990) and the contextual availability theory (Schwanenflugel 1992), we anticipate observing concreteness‐dependent differences in participants' ERP responses. This expectation is particularly relevant to our study design, as the words were presented in isolation. Consequently, participants could not rely on any contextual information during word processing but depend solely on the task demands and their personal semantic knowledge. In line with previous studies (e.g., Gullick et al. 2013; Welcome et al. 2011; West and Holcomb 2000; Xiao et al. 2012; Winsler et al. 2018) and the recent review by Federmeier (2022) revealing that the concreteness effects tend to be more pronounced in tasks emphasizing the processing of semantic information, we expected a more pronounced linear, concreteness effect in the participants' ERP response when the processing of semantic information is required (i.e., in the affective and semantic tasks), especially when an explicit coding of word concreteness is necessary (i.e., in the semantic task). Importantly, we meticulously controlled for potential confounding variables, enabling us to discern the pure effect of concreteness on these neural responses.

Finally, we aimed to investigate not only the fine‐grained modulations of ERP responses by word concreteness (and its interaction with the task demands for semantic processing), as identified through our univariate analyses, but also, for the first time, whether and how the similarity geometry of the ERP spatiotemporal dynamics reflects the neural encoding of concreteness information. To achieve this, we complemented traditional ERP analyses with representational similarity analysis, a multivariate approach that examines the informational content of neural representations. Additionally, we performed an intersection analysis to identify electrodes and timepoints where both univariate ERP concreteness modulations and multivariate neural encoding of concreteness information were present. This step allowed us to integrate the two approaches, providing deeper insights into whether and how the observed ERP concreteness modulations correspond to brain representations directly encoding word concreteness. This integrated framework offers a more comprehensive understanding of the functional significance of ERP concreteness effects.

## 2 Methods

### 2.1 Apparatus and Stimuli

Participants were comfortably seated in a chair in front of a 17‐inch LCD computer monitor (resolution: 1024 × 768 pixels; refresh rate: 60 Hz) at a distance of 60 cm. The presentation of stimuli was controlled by E‐prime software. The experimental stimuli consisted of 176 Italian words derived from Italian affective norms (Montefinese et al. 2013, 2014). The stimuli were selected so that their concreteness values covered as much and as uniformly as possible the entire range of concreteness values. However, abstract and concrete stimuli, as identified based on the median split of their concreteness values, were balanced for a number of lexical‐semantic variables known to affect word processing. In particular, words were balanced for length, frequency, number of orthographic neighbors, and valence, as shown by two‐tailed independent *t‐test* comparisons (all |t|s(174) < 1.571, *p*s > 0.118, Cohen's |d|s < 0.247); moreover, the concreteness values of the selected words had low correlations with the same confounding variables (respectively, *r*s = −0.16, −0.06, 0.11, and 0.04). We also orthogonally manipulated both the grammatical gender (feminine, masculine) and the valence (as the mean value derived from a 9‐point rating scale, 1 = very unpleasant; 9 = very pleasant) of the stimuli. The concreteness values for abstract and concrete words and the valence values for positive and negative words are presented in Figure S1. The mean concreteness values are M = 5.02 (SD = 0.92) for abstract words and M = 7.84 (SD = 0.58) for concrete words, while the mean valence values are M = 2.99 (SD = 0.97) for negative words and M = 7.01 (SD = 0.97) for positive words. All words were presented in Courier New black 24‐point lowercase font on a gray background.

### 2.2 Procedure

**FIGURE 1:**
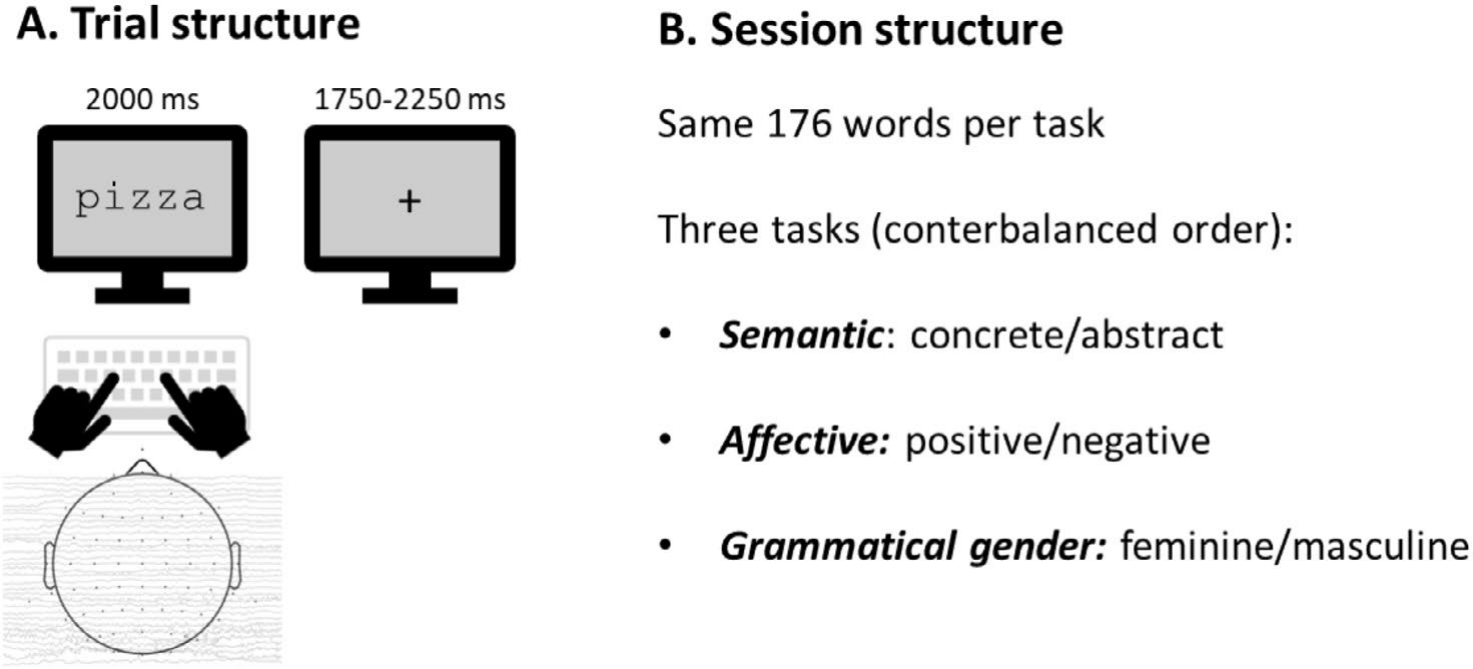
Experimental design structure. (A) Trial structure showing the timing of word presentation (2000 ms) and fixation cross (1750–2250 ms jittered duration). Participants were required to make a dichotomous response to each word as quickly and accurately as possible by pressing one of two keys with their left or right index finger according to the task. (B) Session structure with three tasks (semantic, affective, and grammatical gender decision) administered in counterbalanced order across participants. Each task contained the same set of 176 words.

A summary of the experimental procedure is presented in Figure 1. Each trial included the presentation of a word at the center of the screen for 2000 ms followed by a fixation cross for a mean duration of 2000 ms (range: 1750–2250 ms). On each word, participants were asked to perform three tasks presented in separate blocks. In the semantic task, participants had to judge if the word presented at the center of the screen denoted a concrete or abstract concept. In the affective task, participants had to judge if the word denoted a positive or negative concept. In the grammatical task, participants had to indicate if the word had feminine or masculine grammatical gender. The words were only presented once in each task, but they were the same across the three tasks. Participants were required to respond to each word as quickly and accurately as possible by pressing a key on the keyboard with their left (“F”) and right (“J”) index fingers. Task order and response hands were counterbalanced between participants. Participants completed three tasks of 176 trials each, for 1 h of duration. Participants had a 1‐min rest break every 88 trials and a 3–5 min rest break between each task. A short training session was held to ensure correct task understanding, administering 24 trials that showed words other than those used in the experimental session. During EEG recordings, participants were asked to fixate on the center of the screen to avoid eye movements.

### 2.3 EEG Recording

EEG was recorded from 58 tin electrodes mounted on an elastic cap (ElectroCap) according to the 10–10 International System, and seven additional electrodes applied below each eye (IO1, IO2), in the external canthus of each eye (F9, F10), on the nasion (Nz), and on the mastoids^2^. The EEG signal was recorded with a SynAmps RT amplifier and Curry 7 software (Compumedics Neuroscan, Charlotte, NC, USA), using a sampling rate of 500 Hz, Cz as an online reference, and keeping the electrode impedances below 10 KΩ.

### 2.4 Data Analysis

#### 2.4.1 Behavioral Data

Analyses of RT from the three tasks were performed using linear mixed‐effects models (LMM) implemented with the lme4 library (Bates et al. 2014) in R (http://www.R‐project.org/). Data from the first trial of each task, as well as data from error trials (i.e., incorrect responses in the grammatical task, missing responses, or responses faster than 150 ms) and post‐error trials were not included (mean percentage of excluded trials: 4.01% trials, SD = 3.24% trials). Moreover, to control for the impact of positive skewness in the RT distribution (ms), all the analyses were performed on inverse‐transformed RTs (iRT), computed as −1000/RT (Brysbaert and Stevens 2018).

The specification of the best adequate LMM to test the experimental effects of interest across the three tasks (i.e., Concreteness and its interaction with task) consisted of the following steps. First, several possible confounding predictors that were expected to explain iRT variability were tested using data from the grammatical task (i.e., the baseline task). Specifically, we tested an LMM specified as the following Wilkinson notation formula:

(1) $$\begin{array}{c} \mathrm{iRT}\sim \text{Trial}+\text{PreRT}+n\_\text{letters}\\ +\text{WordFrequency}+n\_\text{OrthographicNeighbours}\\ +\text{freq}\_\text{OrthographicNeighbours}+\mathrm{OLD}20\\ +\text{AgeOfAcquisition}+\left(1|\mathrm{ID}\right)+\left(1|\text{Word}\right) \end{array}$$

In detail, we entered the rank order of each trial (Trial) and the iRT at the preceding trial (PreRT) as fixed‐effect terms to control for the temporal dependencies between successive trials that generally need to be addressed when modeling RTs (Baayen and Milin 2010). Moreover, we added a series of psycholinguistic variables that are known to affect performance in a large variety of cognitive tasks: number of letters (n_letters), word frequency (WordFrequency), number of orthographic neighbors (n_OrthographicNeighbours), mean frequency of orthographic neighbors (freq_OrthographicNeighbours), orthographic Levenshtein distance 20 (OLD20), age of acquisition (AgeOfAcquisition) (for a comprehensive description of these linguistic variables with reference to the Italian language, see, Montefinese, Vinson, et al. 2019). The model also included crossed random intercepts for participants (ID) and word items (Word). In all analyses, continuous variables were centered and scaled within each participant to facilitate model convergence. A model selection procedure from (1) was performed through the function step of the lmerTest R library (Kuznetsova et al. 2017) which performs backward elimination of non‐significant fixed effects of LMM (Kuznetsova et al. 2015). The results of the model selection (see Table S1) revealed that only PreRT, OLD20, and age of acquisition should be retained.

Next, psycholinguistic variables that might represent a confounding factor for the effect of Valence in the affective task were assessed through an LMM on data from the affective task specified as:

(2) iRT~PreRT+OLD20+AgeOfAcquisition+Valence^2+Arousal+Dominance+1ID+1Word

In (2), the linear and quadratic effect of Valence (Valence^2) was tested along with the other two dimensions that compose the emotional space in the dimensional theory of emotion (Osgood et al. 1957), namely Arousal and Dominance. The Valence, Arousal, and Dominance values were derived from the Italian affective norms (Montefinese et al. 2014). The model also included the variables that were retained from (1) (i.e., PreRT, OLD20, and AgeOfAcquisition). The model selection procedure on (2) showed that the inclusion of Dominance was not justified (see Table S2). Although the linear effect of valence was also not significant, it was retained in the model to properly test the quadratic valence effect.

Finally, an LMM on data from the semantic task was fitted to assess the inclusion of possible confounds for Concreteness:

(3) iRT~PreRT+OLD20+AgeOfAcquisition+Valence^2+Arousal+Concreteness^2+Familiarity+Imageability+ContextualAvailability+1ID+1Word

In (3), the linear and quadratic effect of concreteness (Concreteness^2) was tested along with Familiarity, Imageability, and ContextualAvailability^3^ (Montefinese et al. 2014, 2020). Model selection results (see Table S3) showed that the inclusion of Familiarity, Imageability, and ContextualAvailability was not justified.

After the above‐presented three steps, the final LMM to be fitted on data from all the tasks was specified as follows:

(4) iRT~PreRT+OLD20+AgeOfAcquisition+Arousal+Valence^2×Task+Concreteness^2×Task+1ID+1Word

In addition to the confounding variables retained from steps 1–3, the final LMM included the simple effect of Task (three‐level factor with the grammatical task as reference level) and its interactions with Valence^2 and Concreteness^2.

#### 2.4.2 EEG Data Preprocessing

Offline signal preprocessing was performed with MATLAB (Version 2017b; The MathWorks Inc. Natick, MA) using scripts created ad hoc based on the functions from the EEGLAB toolbox (version 14.1.2; Delorme and Makeig 2004). All criteria were established prior to data analysis.

Continuous raw data were filtered offline using zero‐phase Hamming‐windowed sinc FIR high‐pass and low‐pass filters (cut‐off frequencies: 0.1 and 45 Hz, transition bandwidth: 0.2 and 10 Hz. respectively). Moreover, to facilitate the identification and removal of artifacts, we created a temporary cleaner dataset to be submitted to the independent component analysis (ICA) algorithm (Winkler et al. 2015). Specifically, we performed the following temporary pre‐processing steps: (1) we applied a stronger high‐pass filter (cut‐off frequency: 1 Hz, transition bandwidth: 2 Hz); (2) we detected and removed bad channels by means of both the *pop_rejchan* function, using the boxplot method to identify channels with extreme spectral power, and the *clean_rawdata* function, using a correlation threshold of 0.707 (i.e., the correlation corresponding to 50% of shared variance). These criteria led to the exclusion of 1.84 channels on average, SD = 1.73, range = [0–7]. ICA was then performed on this temporary dataset, using the *fastICA* algorithm with a symmetric approach, followed by equivalent dipole fitting performed using *dipfit*. To identify non‐brain components (e.g., eye movements, blinks, and muscular activity), we first used an automatic selection procedure based on an *IClabel* classification probability of being brain IC lower than 50% and a residual variance higher than 20% according to the *dipfit* model. Then, we visually inspected all the components to confirm or modify the automatically flagged ones based on their scalp topography, dipole location, evoked time course, and power spectrum. The ICA solution was applied to the original dataset (after excluding bad channels in it too, so to have the same number of channels in the two datasets) and the previously identified artifactual ICs were rejected, obtaining EEG data cleaned from artifacts detected by ICA.

Subsequently, removed channels were interpolated by using a spherical spline method (Perrin et al. 1989) and data were re‐referenced to a common average reference to provide a reference‐free representation of the data, which is crucial for accurately identifying artifacts. To detect and remove bad epochs, we used a two‐step procedure. We first segmented data into epochs (from −1400 to 2600 ms^4^) with respect to the stimulus onset and we used an automatic procedure to detect artifactual epochs based on extreme values (threshold: ±125 μV), abnormal trend in data (maximal slope allowed = 100 μV/epoch and minimal *R* ^2^ allowed = 0.3), and improbability and kurtosis criteria (for both, SD > 6 for the single‐channel and SD > 4 for the global threshold). We then used the trial‐by‐trial (*TBT*) plugin of EEGLAB, which allowed rejecting and interpolating channels on a TBT basis. Specifically, epochs with more than 6 bad channels were removed whereas, if this criterion was not met, the channels were interpolated. The three criteria together led to the exclusion, on average, of 7.26 epochs (SD = 3.13, range = [1–17]). After these steps, we re‐referenced again all EEG channels to a common average reference (to restore a consistent reference across all channels, taking into account the data interpolated by the TBT procedure) and applied a baseline correction from −200 to 0 ms.

#### 2.4.3 EEG Mass Univariate Analysis

Epochs with RT lower than 150 ms, epochs with incorrect responses in the grammatical task as well as bad epochs identified in the EEG preprocessing phase were excluded from the analysis. Participants' EEG datasets were concatenated, resulting in a final dataset of size 61 channels × 101 timepoints (from 0 to 1000 ms in steps of 10 ms) × 29,529 epochs. The final EEG dataset was analyzed using mass linear mixed‐effects modeling with crossed random effects for participants and words using the lmeEEG procedure (Visalli et al. 2024). The procedure, which enables the use of linear mixed models with crossed random effects in mass univariate analyses of EEG data, overcoming the computational costs of standard available approaches, consisted of three steps. First, for each channel‐timepoint pair we conducted an LMM specified as the following Wilkinson‐notation formula:

(5) $$\begin{array}{c} \mathrm{EEG}\sim \mathrm{iRT}+\mathrm{OLD}20+\text{AgeOfAcquisition}+\text{Arousal}\\ \;+\text{Valence}\times \text{Task}+\text{Concreteness}\\ \;\times \text{Task}+\left(1|\mathrm{ID}\right)+\left(1|\text{Word}\right) \end{array}$$

Specifically, the LMM included all the linguistic variables of the final LMM model for the behavioral analysis^5^. Moreover, iRT (centered and scaled within each participant) was included to account for possible task difficulty effects (Gundel and Wilson 1992). In the second step, random‐effects contributions estimated in the first step were removed from EEG data. At this point, the resulting marginal EEG datasets (i.e., the EEG data cleaned up of the random effects, mEEG) can be analyzed using mass linear regression models (LM). This is the key aspect of lmeEEG. Since LM are extremely faster than LMM, lmeEEG makes permutation testing required to deal with multiple comparisons feasible. In the third step, for each channel‐timepoint pair we conducted an LM specified as the following Wilkinson‐notation formula:

(6) $$\begin{array}{c} m\mathrm{EEG}\sim \mathrm{iRT}+\mathrm{OLD}20+\text{AgeOfAcquisition}\\ \;+\text{Arousal}+\text{Valence}\times \text{Task}+\text{Concreteness}\times \text{Task} \end{array}$$

The threshold‐free cluster enhancement (TFCE) technique (Mensen and Khatami 2013) in conjunction with permutation‐based statistics (5000 permutations, *α* = 0.05) was used to assess the significance of the LM‐parameter effects of interest, namely, the effect of concreteness and its interactions with the task.

#### 2.4.4 EEG Representational Similarity Analysis

To investigate neural representations of concreteness and explore potential task‐dependent influences on them, we performed a representational similarity analysis (Kriegeskorte et al. 2008a) with a spatio‐temporal searchlight approach (Su et al. 2014).

For the first‐level (subject‐specific) analysis, we computed the representational dissimilarity matrix (RDM) of the following variables (model RDMs): Concreteness, Task, AgeOfAcquisition, OLD20, the Valence‐Arousal‐Dominance (VAD) emotion representation (i.e., the 3D coordinates within the VAD space), iRT, text‐based semantic similarity (WEISS, see below), and orthographic similarity (EDIT, see below). Each RDM is a symmetric *n* × *n* matrix, where *n* is the number of analyzed trials (the excluded trials for each participant matched those in the mass univariate analysis) and each off‐diagonal element indicates the distance for each pair of words in a given measure. Specifically, RDMs for Concreteness, AgeOfAcquisition, OLD20, the VAD space, Task, and iRT represented Euclidean distances; the RDM for semantic similarity represented distances taken from the word‐embeddings Italian semantic space (WEISS) model (Marelli 2017), and the RDM for orthographic similarity represented the orthographic edit distance (EDIT), or Levenshtein distance (Yarkoni et al. 2008). Finally, for each channel/timepoint combination, a searchlight region was defined, including neighboring channels (obtained through triangulation using the *ept_ChN2* function from the TFCE toolbox, Mensen and Khatami 2013) and timepoints from −30 to +30 ms. RDMs for the EEG signal from the searchlight region were computed as 1 minus the Pearson's correlation.

Each lower triangular EEG RDM was explained using the following multiple linear regression model:

(7) $$\begin{array}{c} \mathrm{EEG}\;\mathrm{RDM}\sim \mathrm{OLD}20+\text{AgeOfAcquisition}+\mathrm{iRT}\\ \;+\mathrm{VAD}\times \text{Task}+\mathrm{Concreteness}\times \text{Task}\\ \;+\text{WEISS}\times \text{Task}+\text{EDIT} \end{array}$$

Estimated betas for each predictor measure the degree of the representational similarity between model RDMs and EEG RDM (also called representational strength), that is, how strong is the correlation between a given model RDM and the EEG RDM computed from the spatiotemporal pattern of ERPs (at a given electrode and timepoint). It is important here to note that the estimated betas do not have a direct correspondence with fluctuations of EEG signal voltages, as this multivariate approach operates in the representational space reflected by the computed RDMs. This distinction underscores the complementary nature of the two methods: univariate ERP analyses are ideal for identifying spatiotemporal modulations linked to task demands or specific cognitive processes, while multivariate representational similarity analysis provides insights into how features of interest (e.g., concreteness) are encoded in distributed neural patterns.

Second‐level (group‐level) analysis consisted of one‐tailed one‐sample *t*‐tests performed on estimated betas from the first‐level analysis in the channels × timepoints (0–1000 ms) data space. Results for each parameter were corrected for multiple comparisons using the TFCE technique (5000 permutations, *α* = 0.05).

Finally, we performed an intersection analysis to identify the spatiotemporal samples for which there was both a significant encoding of the Concreteness representational model (i.e., the RDM reflecting the similarity in the words Concreteness values) and a significant task‐dependent modulation of the ERP Concreteness effect. This analysis indicates whether the ERP Concreteness effects rely on the brain representation of the word concreteness.

In addition, simple correlations between model RDMs of each single predictor and each participant's EEG‐RDM were computed and tested at the group level as described for the main analysis. Results are presented in Figure S2.

### 2.5 Participants

Fifty‐eight students (43 females; age: M = 20.81 years, SD = 4.14 years; years of education: M = 14.24, SD = 2.03) from the University of Padova participated in the study in exchange for course credits. All participants were right‐handed native Italian speakers with no history of neurological or psychiatric diseases and normal or corrected‐to‐normal visual acuity. They gave informed consent prior to their inclusion in the study, which was approved by the Ethics Committee for Psychological Research of the University of Padova (Protocol number 2945) in accordance with the ethical standards of the Declaration of Helsinki.

While formal power analyses for LMM analyses with crossed random effects are inherently challenging, we adopted a complementary approach to ensure adequate statistical power across both our mass univariate and multivariate representational similarity analyses.

We performed an a priori power analysis in G*Power (Erdfelder et al. 1996) to compute the minimum sample size required to detect, with a statistical power of 0.80, significant effects using a one‐tailed one‐sample t test on the by‐subjects effects (like the beta values estimated in the representational similarity analysis), as this reflects the second step of the regression approach employed in our representational similarity analysis. We assumed a small‐medium Cohen's *d* effect size of at least 0.35, corresponding to a correlation *r* = 0.17. This choice ensured that the sample size was sufficient to detect small‐to‐medium effects in both analytical frameworks. Although this approach does not fully account for the complexity of LMM, it provides a conservative and practical estimate of the minimum sample size required.

Our power analysis revealed that at least 52 participants were required. We nonetheless decided to recruit as many participants as possible, exceeding the required sample size, within the constraints of our available time and resources, to further enhance the robustness and precision of our findings, ultimately achieving a final sample size of 58 participants.

## 3 Results

### 3.1 Behavioral Results

Visual inspection of the full‐model residuals showed that they were skewed. As suggested by Baayen and Milin (2010), trials with absolute standardized residuals higher than 2.5 SD were considered outliers and removed (1.08% of the trials). After the removal of outlier trials, the model was refitted and achieved reasonable closeness to normality.

**FIGURE 2:**
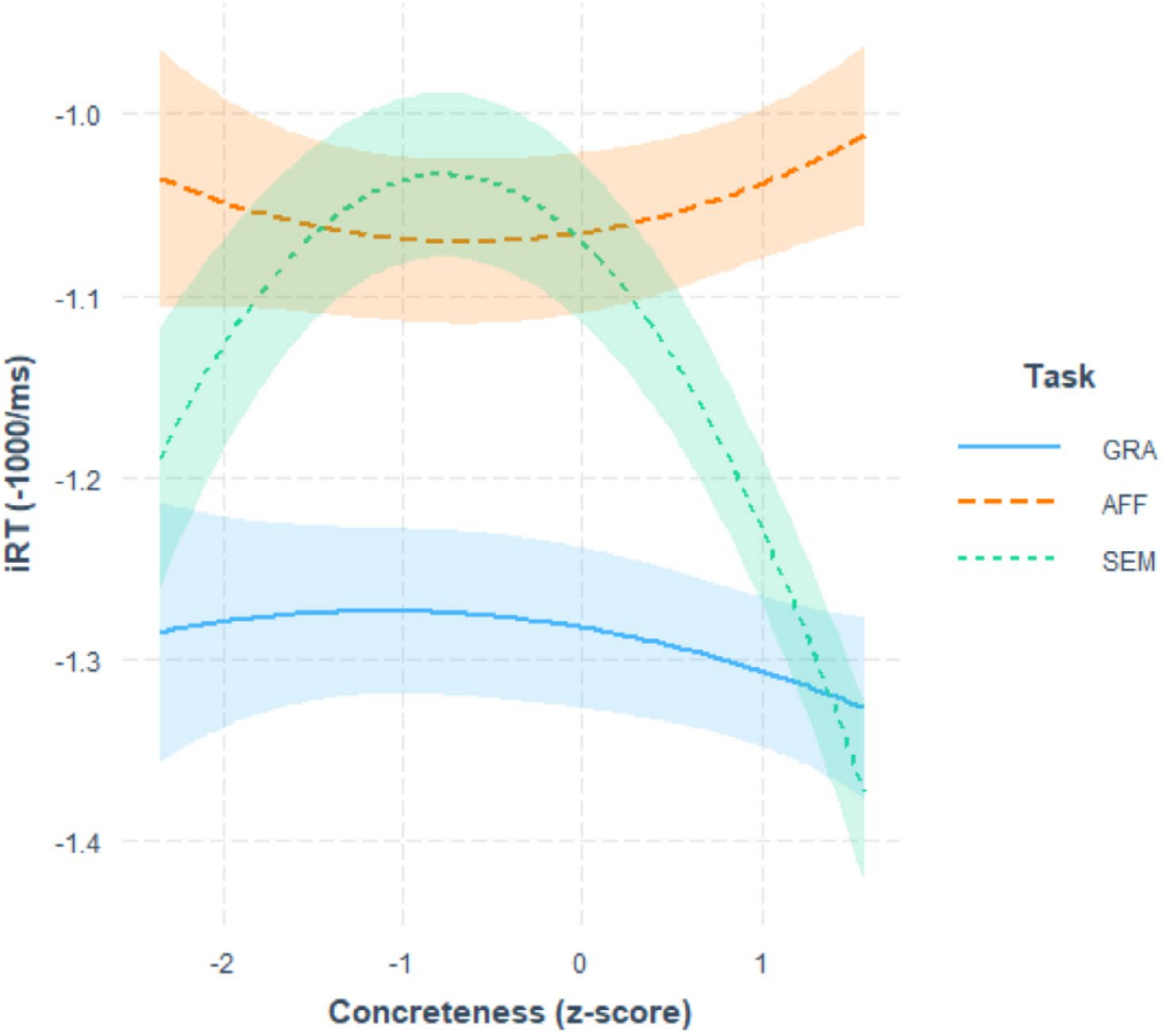
Effects of the interaction between Concreteness and Task. The figure shows the conditional effects of Concreteness (*z*‐scored values) on iRT for the three tasks: Semantic (SEM; in orange dashed line), affective (AFF; green dotted line), and grammatical (GRA; blue continuous line). Shaded error bars indicate 95% confidence intervals.

A summary of the model results is presented in Table S4. Concerning our effects of interest, we found a significant interaction between Concreteness^2 and the contrast between semantic and grammatical tasks (estimate = −0.055, s.e. = 0.005, *t* = −12.07, *p* < 0.001). As shown in Figure 2, participants were faster for extreme Concreteness values in the semantic task. To assess the presence of the quadratic effect of concreteness (Concreteness^2) in the three tasks, the model was refitted two times, changing the reference level of the Task factor. Indeed, the simple effect for Concreteness^2 (as well as for Valence^2) refers to the effect in the reference‐level task. Statistics from the summary outputs confirmed that the effect of Concreteness^2 was significant in the semantic task (estimate = −0.062, s.e. = 0.007, *t* = −9.0, *p* < 0.001), but not in the affective task (estimate = 0.012, s.e. = 0.007, *t* = 1.7, *p* = 0.083), nor in the grammatical task (estimate = −0.007, s.e. = 0.007, *t* = −1.1, *p* = 0.287). In addition to the quadratic effect, Concreteness exhibited a significant simple effect (estimate = −0.016, s.e. = 0.007, *t* = −2.2, *p* = 0.027) as well as significant interactions with the semantic task (estimate = −0.079, s.e. = 0.005, *t* = −17.1, *p* < 0.001) and the affective task (estimate = 0.032, s.e. = 0.005, *t* = 7.0, *p* < 0.001). As shown in Figure 2, participants were slightly faster with higher Concreteness in the grammatical task. Beyond the quadratic effect, this linear effect of Concreteness was greater in the semantic task, while it seems to be reversed in the affective task. The simple effects of Concreteness in the models refitted by changing the Task reference level confirmed that the effect was significant (although in opposite directions) in both the semantic (estimate = 0.096, s.e. = 0.007, *t* = −13.0, *p* < 0.001) and affective (estimate = 0.015, s.e. = 0.007, *t* = 2.1, *p* = 0.037) tasks.

### 3.2 EEG Mass Univariate Analysis Results

**FIGURE 3:**
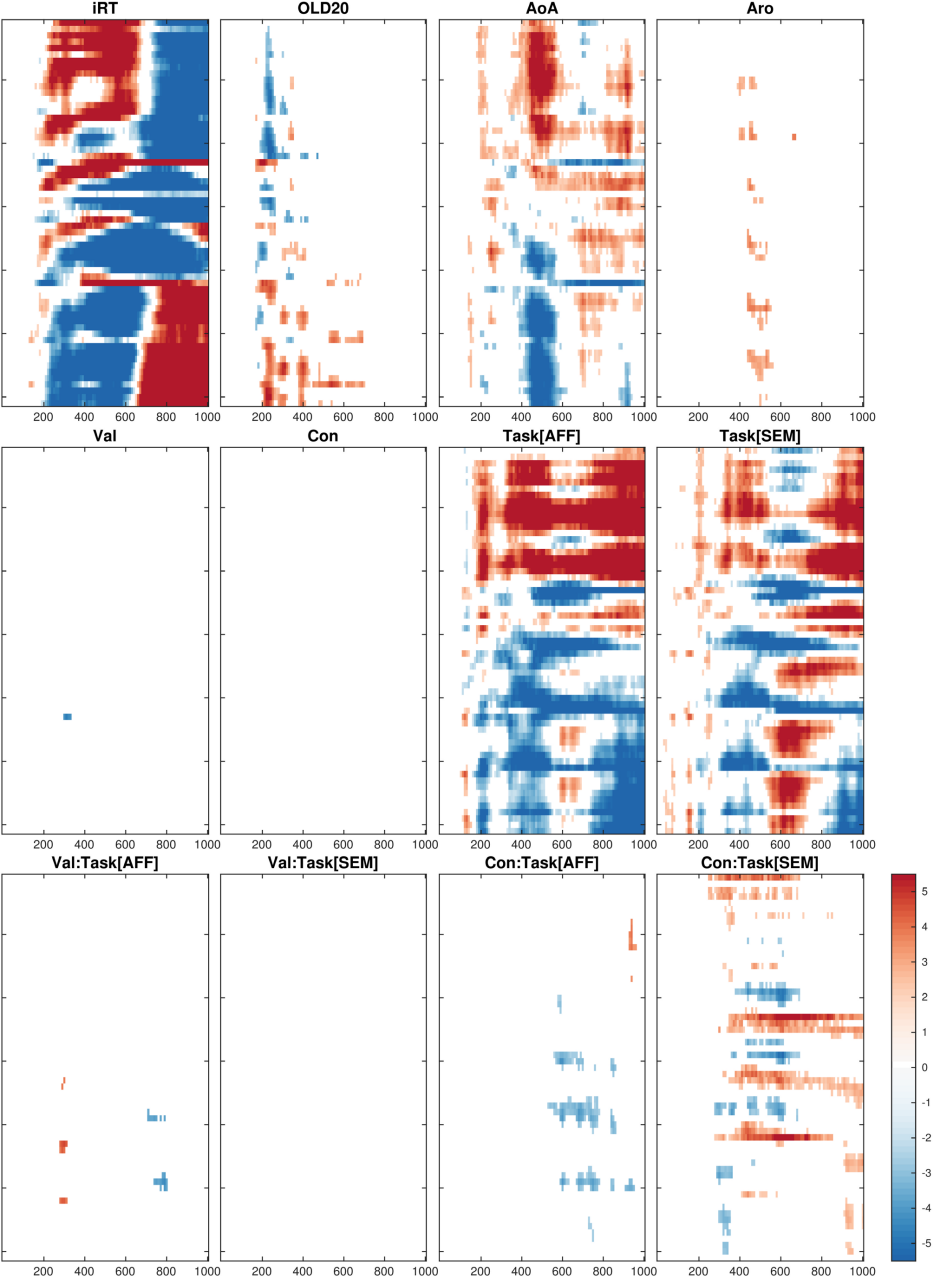
Results of univariate EEG analysis (lmeEEG). The raster diagrams show electrodes/timepoints significantly modulated by the effect indicated in the corresponding heading. White rectangles indicate electrodes/time points for which no significant modulations were observed. Electrodes are on the y axis and are arranged to approximate scalp topography, with top‐to‐bottom corresponding to anterior‐to‐posterior (see Figure 4 for the labels). The x axis represents time in milliseconds from stimulus presentation. The colorbar on the right indicates *t‐*values. “:”, interaction; AoA, age of acquisition; Aro, arousal; Con, concreteness; IRT, inverted response time; OLD20, orthographic Levenshtein distance 20; Task[AFF], contrast between the grammatical and the affective tasks; Task[SEM], contrast between the grammatical and the semantic tasks; Val, valence.

**FIGURE 4:**
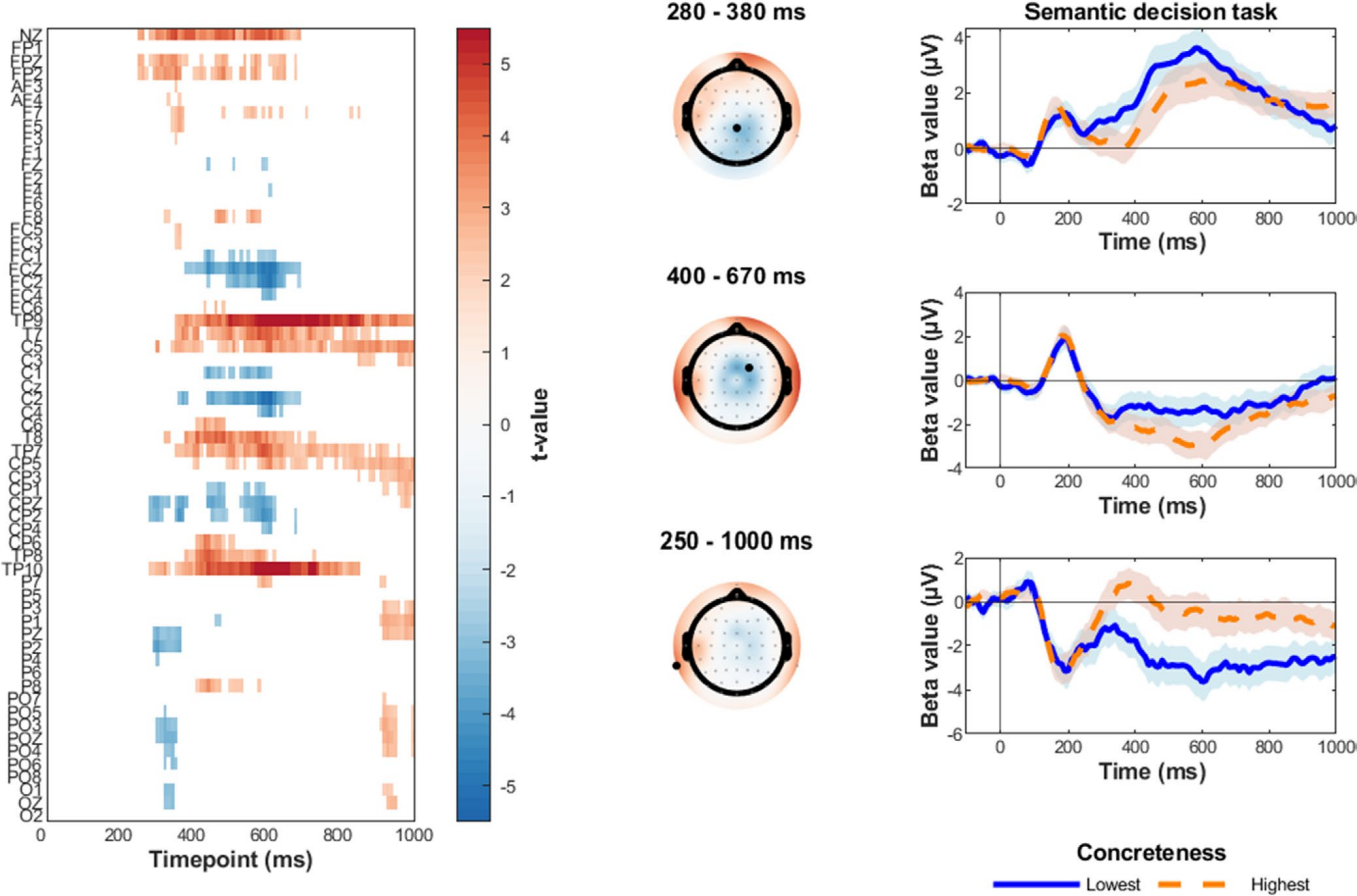
Significant effects elicited by the Concreteness ×Task[SEM] interaction in target‐locked EEG. The raster diagram (left) shows electrodes/timepoints significantly modulated by the interaction. White rectangles indicate electrodes/time points for which no significant modulations were observed. The topoplots (middle panels) show the *t* values (same color scale as the raster diagram) averaged in the indicated time windows. The trace‐plots (right panels) depict the LMM estimated responses for the highest (orange dotted line) and lowest (blue solid line) concreteness in the semantic task for the electrodes indicated as black dots in the corresponding topoplots (i.e., CPz, FC2, and TP9, respectively). Error bands indicate 95% confidence intervals.

Results of the EEG mass univariate analysis (lmeEEG) are presented in Figure 3. Concerning the simple effect of Concreteness (i.e., its effect in the grammatical task), we did not find any significant EEG modulation. However, looking at the interaction between Concreteness and Task variables, we found significant modulations in the semantic task (Figure 4). Specifically, we observed three significant clusters.

The first cluster emerged over parietal electrodes between 280 and 380 ms and was characterized by a more pronounced negativity (akin to the N2 ERP component) followed by a less pronounced positivity (akin to the P3 ERP component) with higher Concreteness values. The second cluster was found over fronto‐central electrodes in a time window ranging from 400 to 670 ms. It was characterized by a more pronounced negativity (akin to the N400 ERP component) with higher Concreteness values. The third cluster consisted of a more sustained negativity over bilateral fronto‐temporal electrodes starting from 250 ms with lower Concreteness values.

**FIGURE 5:**
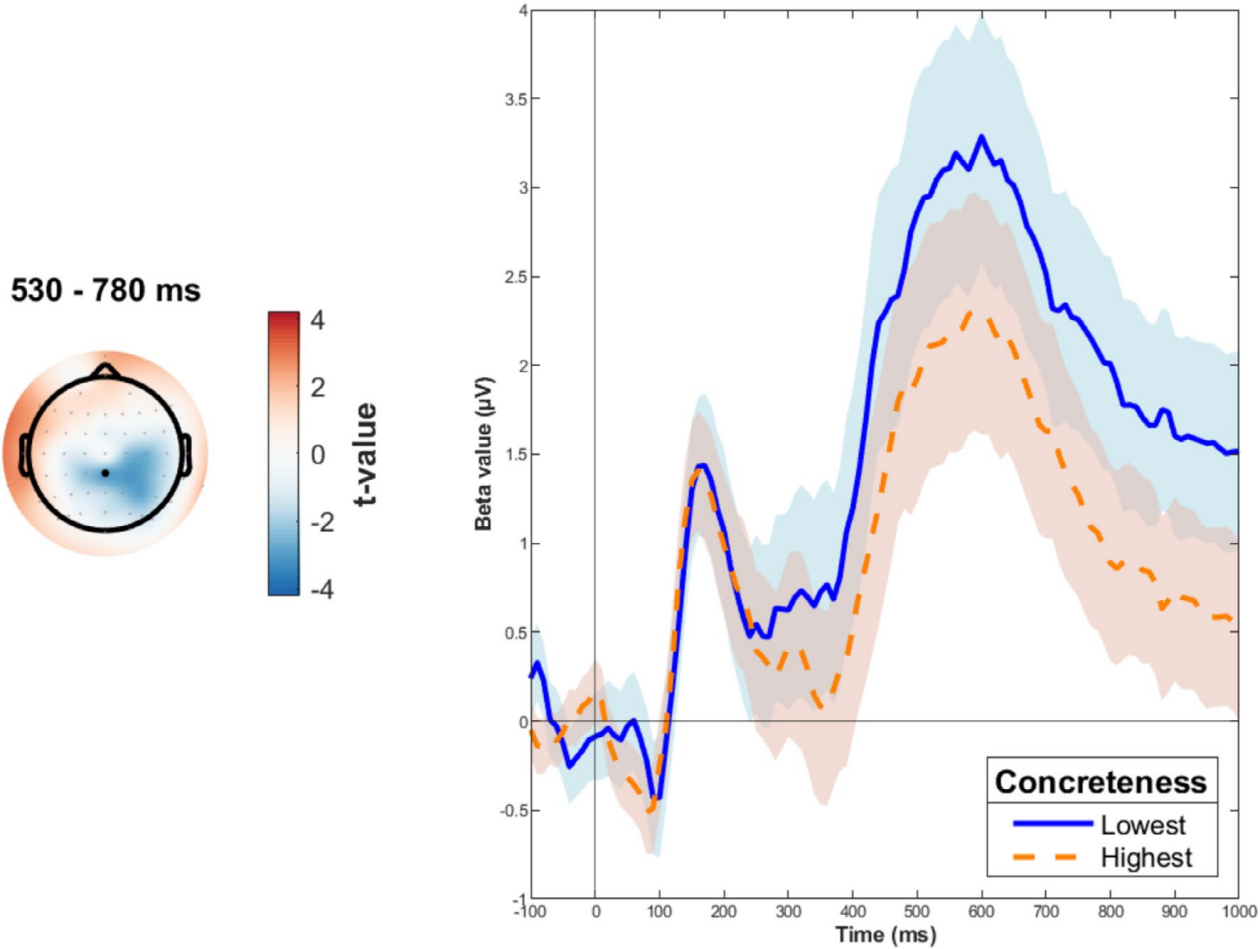
Significant effect elicited by the Concreteness × Task[AFF] interaction in target‐locked EEG. The topoplot (left panel) shows the *t* values averaged in the indicated time windows. The trace‐plot (right panel) depicts the LMM estimated responses for highest (orange dotted line) and lowest (blue solid line) Concreteness values in the valence task for the CPz electrode indicated as a black dot in the topoplot. Error bands indicate 95% confidence intervals.

We also observed an effect of Concreteness in the affective task that differed from the one observed in the semantic task. Indeed, it was characterized by a more pronounced positivity (akin to a P600 ERP component) with lower Concreteness values over centroparietal electrodes in a time window ranging from 530 to 780 ms (Figure 5).

### 3.3 EEG Representational Similarity Analysis Results

**FIGURE 6:**
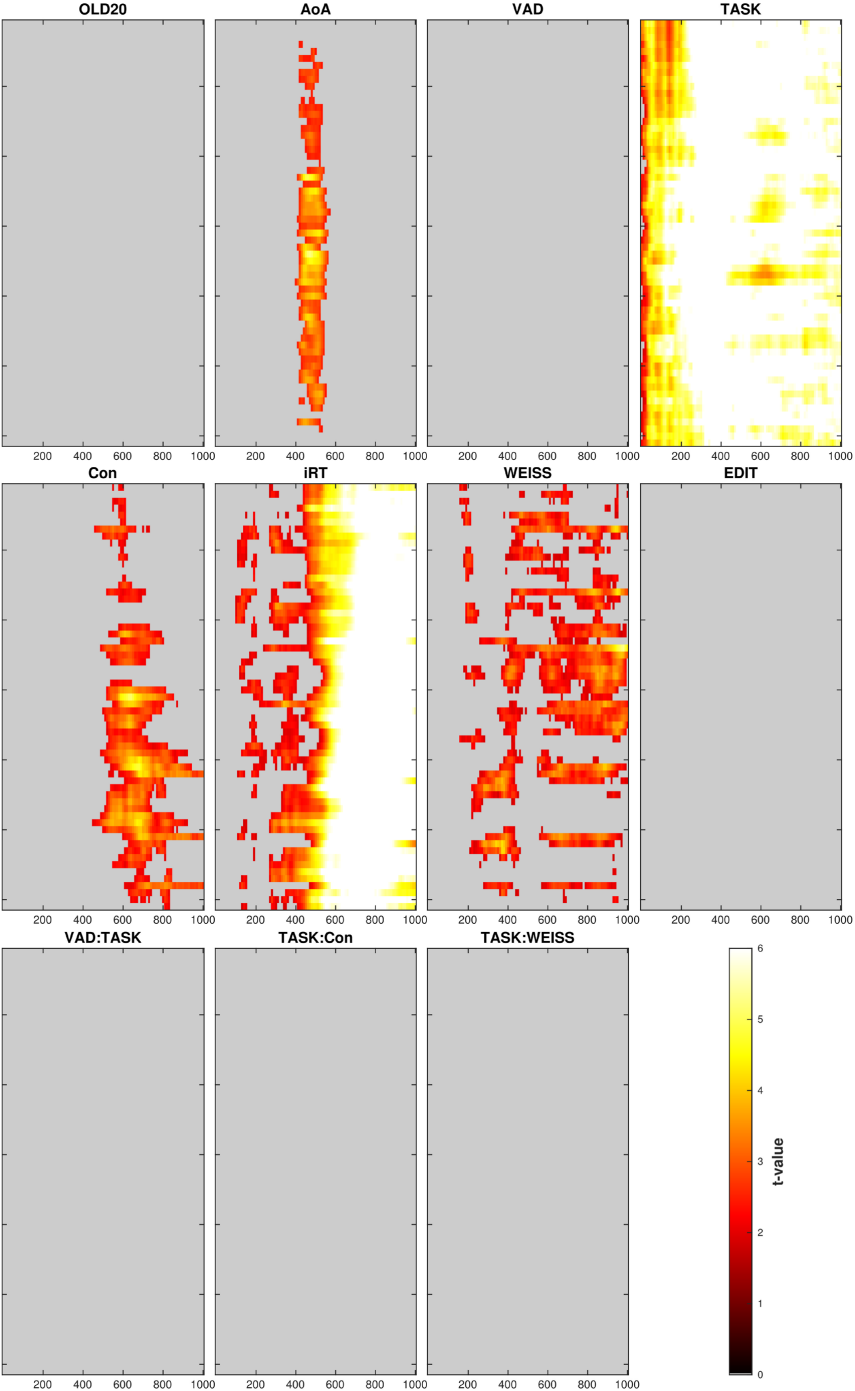
Representational similarity analysis results. The raster diagrams show electrodes/timepoints with significant EEG‐model representational similarities for the model indicated in the corresponding heading. Gray rectangles indicate electrodes/time points for which no significant EEG‐model representational similarities were observed. VAD, Valence‐Arousal‐Dominance space; WEISS, word‐embeddings Italian semantic space; Levenshtein distance for orthographic similarity; see Figure 3 for other conventions.

Results of the representational similarity analysis are presented in Figure 6. Concerning our effects of interest, we found significant representational similarities for Concreteness starting from 450 ms with a widespread scalp distribution. Moreover, we did not find any significant interaction between Concreteness and Task, which indicates that there was not a significant difference for Concreteness representational similarities across tasks.

**FIGURE 7:**
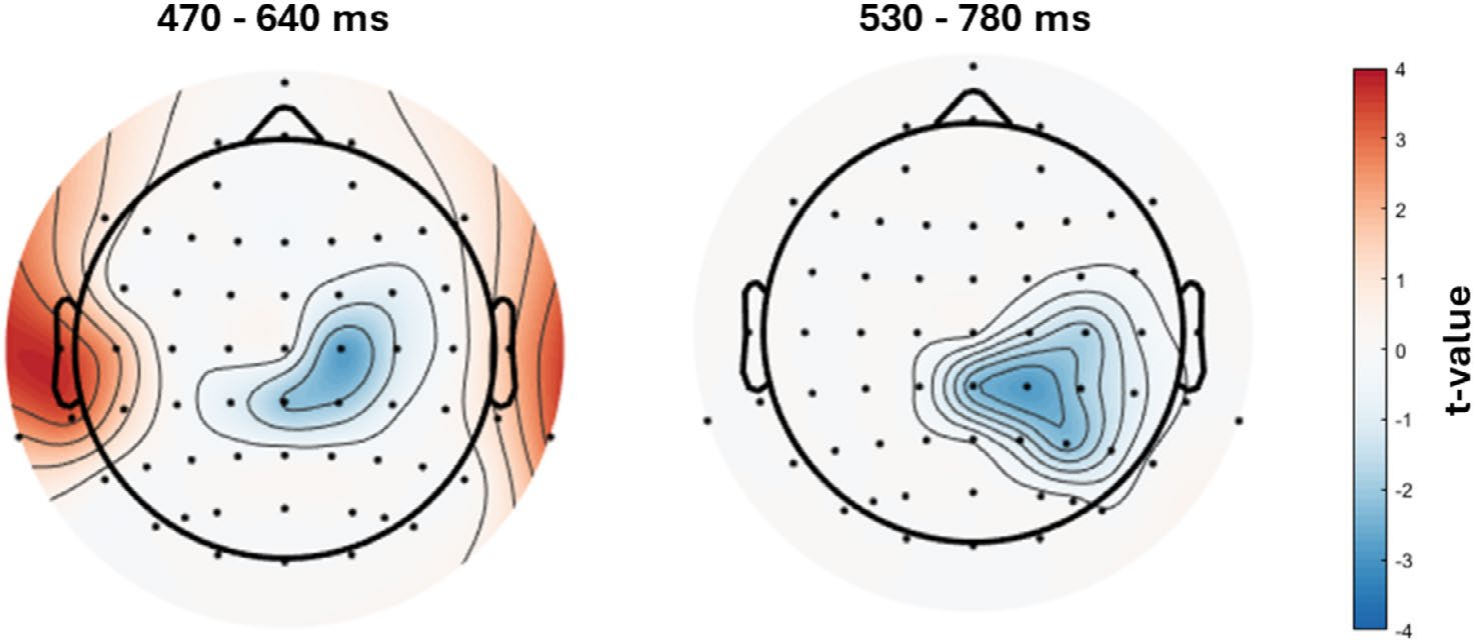
Intersection analysis results. The topoplots show the *t* values of the significant ERP modulations reflecting the Concreteness × Task[SEM] interaction (left panel) and the Concreteness × Task[AFF] interaction as revealed by the mass univariate analysis, which were masked by the significant encoding of the Concreteness values revealed by the multivariate representational similarity analysis, averaged in the indicated time windows.

Figure 7 presents the results of the intersection analysis, which reflect the cognitive processes underlying word processing that are grounded on brain representations based on word concreteness informational content. This analysis first revealed the electrodes and time window where we observed significant fine‐grained modulations of ERP responses by word Concreteness in the semantic task, masked by the electrodes/timepoints where we also observed significant neural encoding of concreteness information (Figure 7, left panel; cfr. Figure 4). Significant results were observed for most of the electrodes/timepoints belonging to both the central cluster showing the Concreteness modulation of the N400‐like component and the bilateral temporal cluster showing the Concreteness modulation of the ERP sustained negativity. By contrast, since the representational similarity results were only significant after 450 ms, the intersection analysis did not reveal significant electrodes/timepoints belonging to the earlier posterior cluster showing the Concreteness modulation of the N2/P3‐like component.

This analysis also revealed the electrodes and time window where we observed significant fine‐grained modulations of ERP responses by word Concreteness in the affective task, masked by the same electrodes/timepoints where we also observed significant Concreteness encoding (Figure 7, right panel; cfr. Figure 5). Significant results were observed for most of the electrodes/timepoints belonging to the centroparietal cluster showing the Concreteness modulation of the P600‐like component.

## 4 Discussion

In this study, we investigated how word concreteness, treated as a continuous measure, affects word processing, as posited by theoretical accounts of the concreteness effect (Paivio 1990; Schwanenflugel et al. 1992; Barber et al. 2013). Furthermore, we examined whether and how this concreteness effect is modulated by the task demands, employing three tasks with varying requirements for semantic processing.

At the behavioral level, results showed that concreteness affected word processing differently depending on the task, in line with our hypotheses. Indeed, in the semantic task, which directly required participants to access semantic information regarding the word concreteness, we observed faster reaction times for words at both lower and higher concreteness values, reflecting the easier semantic decision for these stimuli. In the same task, we also identified a linear concreteness effect, in line with literature demonstrating faster reaction times for more concrete words (Bleasdale 1987; Kroll and Merves 1986; Miller and Roodenrys 2009; Romani et al. 2008). Crucially, this linear concreteness effect was modulated by the task. Indeed, it was smaller and not significant in the grammatical task, in which processing word semantic information was task‐irrelevant. Notably, in the affective task, which ensured access to semantic representation and, indirectly, to word concreteness, a reverse effect emerged, with faster reaction times for more abstract words. This finding fits well with the notion that abstract meanings evoke more inner and interoceptive sensations and are often linked to heightened emotional experiences (Kousta et al. 2011; Newcombe et al. 2012; Ponari et al. 2018; Vigliocco et al. 2014) compared to concrete concepts (Connell et al. 2018). Interestingly, despite the fact that we balanced abstract and concrete words for their valence, in our dataset there was a non‐negligible correlation between concreteness and arousal (*r* = −0.28, *p* = 0.002), with more abstract words related to stronger emotional responses. Therefore, it is not surprising that we found a small processing advantage for more abstract concepts in the emotion‐related task.

More importantly, we investigated the spatiotemporal dynamics of the fine‐grained effect of concreteness on ERP data, and its modulation by the task requirements, by employing mass linear mixed‐effects modeling with crossed random effects for both participants and words. As expected, we generally observed linear concreteness effects on ERP responses that varied with the task and changed over time and brain regions. In particular, in the semantic task (focusing on word meanings), we found three spatiotemporal clusters showing ERP concreteness effects. In this task, as predicted, more concrete words showed greater central ERP negative deflections within the N400 time range. Moreover, more abstract words showed a higher parietal positivity within the N2/P3 time range, and more abstract words elicited increased sustained ERP negative deflections in the left temporo‐lateral area. By contrast, in the affective task, more abstract words elicited a more pronounced parietal P600‐like ERP component.

These task‐dependent modulations of the ERP concreteness effects indicate that they emerge mainly when participants are processing word meanings, supporting previous research. In sum, our ERP results partially confirm our initial hypotheses regarding the effects of concreteness and their task‐dependent modulations. Contrary to our expectations, based on the dual‐coding theory (Paivio 1990) and the context availability theory (Schwanenflugel et al. 1992), we found some ERP effects that do not correspond to the classic ERP components reported in the literature (e.g., Adorni and Proverbio 2012; Barber et al. 2013; Bechtold et al. 2022; West and Holcomb 2000). In the following sections, we will discuss each of these findings and their theoretical implications in detail.

Finally, using representational similarity analysis, we found that the word concreteness was significantly encoded by ERP spatiotemporal patterns starting from 450 ms onwards with a widespread bilateral scalp distribution, regardless of the task, indicating that the brain organizes word meanings based on their concreteness.

### 4.1 Fine‐Grained Concreteness Effect in the N2/P3‐Like Component

In our study, we found that more abstract words determined an increased parietal P3‐like component in the semantic task as compared to both the affective and the grammatical tasks. This might reflect the higher working memory demands in deciding the concreteness of more abstract words, in line with the proposed role of the parietal P3 in working memory updating (Courchesne et al. 1975; Dien et al. 2004; Donchin 1981; Friedman et al. 2001; Goldstein et al. 2002). Indeed, abstract words are more complex because they can have many meanings and their level of abstractness can vary (Montefinese et al. 2023). Selecting the correct meaning of more abstract words from semantic memory and subsequently estimating its degree of concreteness becomes a more intricate task, demanding increased resources from working memory. In contrast, more concrete words, with their fewer meanings and less variability in concreteness, would impose a comparatively lighter load on working memory when judging their concreteness. Our interpretation is in line with the intriguing proposition that the decision‐making framework, characterized by gradual evidence accumulation, extends beyond mere sensory sampling to encompass the retrieval of internal memory representations (van Ede and Nobre 2023). This interpretation of the P3‐like concreteness effect we observed also explains why this effect was specifically observed in the semantic task. Indeed, deciding if a more abstract word denotes a positive or negative concept or, especially, if it has a feminine or masculine grammatical gender would not necessarily require the same high working memory demands.

Our results are also consistent with the locus coeruleus‐P3 model (Kok 2001; Kutas et al. 1977), which proposes that the P3 reflects a selective response of the locus coeruleus norepinephrine system to motivationally significant stimuli, particularly those that are task‐relevant. According to this model, the P3 response would reflect the processes required to evaluate and categorize stimuli as targets or non‐targets. Again, the higher variability in meanings and concreteness levels of more abstract words would engage more of these processes, especially when deciding about their concreteness level in the semantic task.

However, a note of caution is essential in interpreting our findings as a P3‐like modulation. Indeed, the spatiotemporal dynamics of this ERP concreteness effect were not related to the neural encoding of the word concreteness, as revealed by the intersection analysis with the representational similarity results. This suggests that while the modulation is more pronounced in the semantic task, where participants explicitly judged the concreteness of words, it may not directly reflect representational processes encoding concreteness per se. This challenges the P3‐based interpretation, which relies heavily on the assumption that the observed modulation reflects working memory or decision‐making processes grounded in concreteness representations. Moreover, the effect we observed was mainly significant at the beginning of the P3‐like component, further raising the possibility that it could instead reflect a more pronounced N2‐like component for more concrete words when performing the semantic task. Previous studies have indeed linked the posterior N2‐like component to visual attention and the processing of salient stimuli (Aine and Harter 1984; Keil and Müller 2010; Kok 2001). Concrete words, with their stronger reliance on visual imagery, as suggested by the dual‐coding theory (Paivio 1990), might attract more attention during early stages of processing, particularly in tasks explicitly requiring semantic judgments.

Nonetheless, our data do not allow us to conclusively attribute the observed effect to one interpretation over the other. However, our findings strongly indicate that, as expected, the brain processes abstract and concrete words differently when evaluating their degree of concreteness, and that this differentiation emerges at a relatively early stage of word processing.

### 4.2 Fine‐Grained Concreteness Effect in the N400‐Like Component

The concreteness effect on the N400‐like component we observed in our study provides valuable insights about how the brain processes words with different levels of concreteness. This ERP response, peaking around 400 ms after word presentation, is closely associated with semantic processing (Bechtold et al. 2023). Consistent with previous research, we found a stronger N400 response for concrete words compared to abstract words (e.g., Bechtold et al. 2022; Barber et al. 2013; Welcome et al. 2011; West and Holcomb 2000). This difference is often explained by the context availability theory (Schwanenflugel et al. 1992), which posits that concrete words elicit larger N400 amplitudes because they easily evoke related contextual information, even when presented in isolation. Indeed, we observed the N400 concreteness effect primarily when explicit semantic processing is required. However, the topographical distribution of our detected cluster does not fully align with the typical centroparietal distribution associated with the standard N400 concreteness effect. Instead, we observed a slightly more anterior distribution. This suggests that our results might also reflect the stronger semantic activation for concrete words, possibly due to the integration of the multimodal features characterizing their richer semantic networks (Barber et al. 2013; Amsel 2011; Rabovsky et al. 2012).

Our innovative approach of treating concreteness as a continuous measure, rather than a dichotomous category, allowed us to observe gradual changes in brain ERP responses as word concreteness varied. Importantly, this effect was specifically present when participants were explicitly asked to consider word concreteness to perform our semantic task, suggesting that the concreteness‐dependent modulation of the N400 response is primarily elicited during active semantic processing. This finding appears to contradict previous literature that reported N400 concreteness effects in tasks requiring implicit semantic processing, such as lexical decision tasks (e.g., Barber et al. 2013; Kanske and Kotz 2007; Kounios and Holcomb 1994). We propose that this discrepancy might be due to our use of continuous measures, which accounts for certain confounding variables (e.g., age of acquisition) that are often collinear with concreteness in dichotomous approaches. As demonstrated in Figure S3, when these confounding variables are not controlled for, N400 responses to concreteness are present even in the grammatical task. This study's consideration of confounding variables has potentially revealed effects that previous research may have overlooked. Furthermore, the practice of converting continuous variables into binary categories typically results in two significant drawbacks. First, it tends to underestimate the true size of effects. Second, it reduces the statistical power of hypothesis tests, making it harder to detect genuine effects (e.g., Cohen 1983).

Contrary to our expectations, we did not observe the N700 concreteness effect. This divergence from previous findings might be attributed to the task‐sensitive nature of the N700, which is typically larger in tasks requiring engagement in imagery processes (Gullick et al. 2013; West and Holcomb 2000). These findings align with theories like the dual‐coding account (Paivio 1990), which attribute concreteness effects to the availability of perceptual and/or motor imagery primarily or exclusively for concrete words. The absence of the N700 effect in our study suggests that, after controlling for confounding variables, our semantic task may not heavily rely on perceptual and motor imagery processes.

### 4.3 Fine‐Grained Concreteness Effect in the Bilateral Fronto‐Temporal Sustained Negativity Component

In our study, we found that more abstract words elicited a more pronounced sustained negativity. This effect was strongest over the temporo‐lateral region, exhibiting a strong left‐lateralized distribution and extending to the fronto‐lateral electrodes. This pattern emerged as early as 250 ms following stimulus onset. This suggests that, as predicted, the brain processes less concrete words differently from more concrete words when participants are making decisions that, as in our affective task, require accessing the word meanings. Our findings regarding the engagement of the temporo‐lateral electrodes appear to be in line with previous research suggesting their role in lexico‐semantic processing (Binder et al. 2009; Sabsevitz et al. 2005). The observed activation of these scalp regions during the processing of more abstract words might thus indicate a potential increased involvement of the verbal semantic system, particularly when compared to the processing of more concrete words.

The significant concreteness effect we found in the affective task extended over bilateral frontal regions of the scalp. This might be due to the distinctive modality of acquiring abstract concepts (Borghi et al. 2017; Montefinese 2019). We usually learn concrete concepts through our senses, by interacting with the physical world, but we learn abstract concepts mainly through language, by hearing or reading them in different contexts. This result also aligns with what we know about the role of frontal regions in conditions with high demands of semantic control (Jefferies 2013; Montefinese et al. 2020; Montefinese, Hallam, et al. 2021; Lambon Ralph et al. 2017). Processing more abstract words is assumed to require more demands of semantic control because it is harder to access the meaning of abstract words from the semantic representation. Indeed, as already noted, abstract words often have multiple meanings, possibly with varying degrees of concreteness, and the brain needs to choose the right one based on the context and task. In contrast, concrete words are closely tied to real‐world objects and usually have a clear, stable meaning. This interpretation aligns with an ERP study by Mondini et al. (2008) comparing countable and mass nouns. Mass nouns, despite being concrete, showed lower concreteness and imageability, with slower RT in semantic categorization tasks. Morphosyntactically, mass nouns resemble abstract words, lacking plural forms and associating with indefinite quantifiers. The study revealed a left fronto‐temporal negativity to mass nouns in the 400–500 ms interval, similar to the greater left temporal‐lateral negativity observed for abstract words in our study. These findings support the view of concreteness as a continuum, even within concrete words, with less concrete items (mass nouns) eliciting processing patterns similar to abstract words.

However, since to the best of our knowledge our results are novel in the ERP literature, further and more direct research (e.g., source localization techniques) would be needed to confirm our interpretations.

### 4.4 Fine‐Grained Concreteness Effect in the P600‐Like Component

We also found that concreteness affected brain activity in the affective task. More abstract words elicited a stronger parietal P600‐like component for more abstract words. This interesting result fits with the idea that abstract words have more emotional content than concrete words (Connell et al. 2018; Montefinese et al. 2020), as also reflected in our dataset (see above). Previous studies have linked the P600‐like component to embodiment effects, particularly in the context of metaphoric language, wherein larger positive deflections have been observed (Forgács et al. 2015; Muraki et al. 2020). Based on this, the concreteness modulation of the P600‐like component we observed may indeed reflect embodiment effects for more abstract words. This interpretation comes from the theory that abstract concepts rely on the interoception modality, as explained by Connell et al. (2018). Interoception involves the processing of sensations within the body and is closely tied to the embodiment of abstract concepts. Importantly, this effect specifically emerged in the affective task. This matches what we found in participants' behavior, as they were better at processing more abstract concepts in the affective task.

### 4.5 Concreteness as an Organizational Principle in the Brain

Our mass univariate analyses revealed important differences in how words are processed based on their concreteness. We used a multivariate analytical method called representational similarity analysis to investigate how the similarity geometry based on word concreteness relates to neural activity patterns. This innovative approach helped us understand the complex relationship between concreteness and neural responses, giving us insights into how words are represented in the brain along the concreteness scale, and whether and how this neural representation is used by downstream cognitive processes involved in word understanding.

This method has been useful in neuroimaging research, looking at how brain responses relate to the meaning of concepts. Most previous studies focused on concrete concepts (Devereux et al. 2018; Fairhall and Caramazza 2013; Kriegeskorte et al. 2008b) with some exceptions looking at abstract concepts (e.g., Wang et al. 2018). Recently, researchers have used this method to study differences between abstract and concrete concepts, employing various similarity measures of meaning (Meersmans et al. 2020; Montefinese, Pinti, et al. 2021). However, until now, no study has clearly shown that concreteness, as a continuous feature of concepts, is represented in our brain.

Our findings show that the brain represents concreteness in detail, even after accounting for other confounding factors, consistently across different tasks. This suggests that concreteness is not only important in how words are processed, but also in how meanings are organized in the brain. Importantly, the intersection analysis revealed that the concreteness effects we observed in the semantic task in both the N400 and the bilateral fronto‐temporal sustained negativity components align with timepoints and scalp regions where word concreteness was significantly encoded by the spatiotemporal pattern of ERP activity. The same was true for the concreteness effect we observed in the affective task in the P600 component. This suggests that these ERP modulations may reflect cognitive processes that actively rely on the neural representation of concreteness, such as semantic integration (N400), prolonged lexical‐semantic processing (sustained negativity), and embodiment effects (P600). Overall, these results highlight the important role of concreteness in how meanings are represented in the brain and in how these neural representations aid word processing.

## 5 Conclusion

To sum up, our study provides novel insights into how word concreteness, treated as a continuous measure, affects word processing and its neural correlates. Our findings demonstrate that the concreteness effect is modulated by task demands and is reflected in distinct spatiotemporal ERP patterns. The observed task‐dependent modulations in behavioral and ERP responses challenge existing theoretical frameworks such as the dual‐coding theory (Paivio 1990) and the context availability theory (Schwanenflugel et al. 1992), suggesting the need for a re‐examination of existing theories on the concreteness effect and its neural correlates. Our results suggest a more nuanced understanding of how the brain processes words along the concreteness continuum, particularly in terms of working memory demands, semantic activation, and emotional content.

Furthermore, our use of multivariate representational similarity analysis, followed by an intersection analysis with univariate results, revealed that word concreteness serves as an organizational principle in the brain, consistently encoded by neural patterns across different tasks, and affects downstream word processing and univariate ERP concreteness effects. This finding extends our understanding of how semantic knowledge is represented and processed in the brain.

These results underscore the importance of treating concreteness as a continuous variable and considering task context when studying word processing. Our approach, made possible by advanced computing power and analytical techniques (like representational similarity analysis and linear mixed‐effects models), offers a more comprehensive view of the concreteness effect than previous dichotomous comparisons. Future research should further explore the implications of these findings for theories of semantic processing and investigate how other semantic dimensions interact with concreteness in shaping word representations in the brain.

## Author Contributions

**Maria Montefinese:** conceptualization, data curation, formal analysis, funding acquisition, investigation, methodology, supervision, writing – original draft, writing – review and editing. **Antonino Visalli:** data curation, formal analysis, writing – original draft, writing – review and editing. **Alessandro Angrilli:** funding acquisition, resources, software, supervision, writing – review and editing. **Ettore Ambrosini:** conceptualization, data curation, formal analysis, funding acquisition, methodology, resources, supervision, writing – original draft, writing – review and editing.

## Conflicts of Interest

The authors declare no conflicts of interest.

## Supporting information


Data S1.

## Data Availability

Data available on request from the authors.

## Endnotes

^1^
This technique assesses whether the similarity structures are analogous across various representational domains, by means of a one‐to‐one mapping between representational geometries from behavioral measurement or computational modeling and neurophysiological measurements.

^2^
The electrode positions recorded from were: AF3 (X: 76.15, Y: 31.48, Z: 20.85); AF4 (X: 76.15, Y: −31.48, Z: 20.85); C1 (X: 0.00, Y: 34.54, Z: 77.67); C2 (X: 0.00, Y: −34.61, Z: 77.64); C3 (X: 0.00, Y: 63.17, Z: 56.87); C4 (X: 0.00, Y: −63.17, Z: 56.88); C5 (X: 0.00, Y: 80.83, Z: 26.29); C6 (X: 0.00, Y: −80.83, Z: 26.29); CP1 (X: −32.44, Y: 32.35, Z: 71.60); CP2 (X: −32.44, Y: −32.35, Z: 71.60); CP3 (X: −30.96, Y: 59.27, Z: 52.47); CP4 (X: −30.96, Y: −59.27, Z: 52.47); CP5 (X: −28.76, Y: 76.25, Z: 24.17); CP6 (X: −28.76, Y: −76.25, Z: 24.17); CPZ (X: −32.93, Y: −0.00, Z: 78.36); Cz (X: 0.00, Y: 0.00, Z: 85.00); F1 (X: 59.91, Y: 26.04, Z: 54.38); F2 (X: 59.87, Y: −26.03, Z: 54.43); F3 (X: 57.55, Y: 48.20, Z: 39.87); F4 (X: 57.58, Y: −48.14, Z: 39.89); F5 (X: 54.04, Y: 63.06, Z: 18.13); F6 (X: 54.03, Y: −63.04, Z: 18.21); F7 (X: 49.87, Y: 68.42, Z: −7.49); F8 (X: 49.93, Y: −68.38, Z: −7.49); FC1 (X: 32.44, Y: 32.35, Z: 71.60); FC2 (X: 32.44, Y: −32.35, Z: 71.60); FC3 (X: 30.96, Y: 59.27, Z: 52.47); FC4 (X: 30.96, Y: −59.27, Z: 52.47); FC5 (X: 28.76, Y: 76.25, Z: 24.17); FC6 (X: 28.76, Y: −76.25, Z: 24.17); FCZ (X: 32.93, Y: −0.00, Z: 78.36); FP1 (X: 80.78, Y: 26.13, Z: −4.00); FP2 (X: 80.78, Y: −26.13, Z: −4.00); FPZ (X: 84.98, Y: −0.00, Z: −1.79); FZ (X: 60.74, Y: −0.00, Z: 59.46); NZ (X: 77.63, Y: −0.00, Z: −34.61); O1 (X: −80.78, Y: 26.13, Z: −4.00); O2 (X: −80.78, Y: −26.13, Z: −4.00); OZ (X: −84.98, Y: −0.00, Z: −1.79); P1 (X: −59.91, Y: 26.04, Z: 54.38); P2 (X: −59.87, Y: −26.03, Z: 54.43); P3 (X: −57.55, Y: 48.20, Z: 39.87); P4 (X: −57.58, Y: −48.14, Z: 39.89); P5 (X: −54.04, Y: 63.06, Z: 18.13); P6 (X: −54.03, Y: −63.04, Z: 18.21); P7 (X: −49.87, Y: 68.42, Z: −7.49); P8 (X: −49.93, Y: −68.38, Z: −7.49); PO3 (X: −76.15, Y: 31.48, Z: 20.85); PO4 (X: −76.15, Y: −31.48, Z: 20.85); PO5 (X: −72.80, Y: 42.95, Z: 8.93); PO6 (X: −72.80, Y: −42.95, Z: 8.93); PO7 (X: −68.69, Y: 49.71, Z: −5.96); PO8 (X: −68.72, Y: −49.67, Z: −5.95); POZ (X: −79.03, Y: −0.00, Z: 31.30); PZ (X: −60.74, Y: −0.00, Z: 59.46); T7 (X: 0.00, Y: 84.54, Z: −8.85); T8 (X: 0.00, Y: −84.54, Z: −8.85); TP10 (X: −23.30, Y: −70.08, Z: −42.09); TP7 (X: −26.21, Y: 80.41, Z: −8.51); TP8 (X: −26.28, Y: −80.39, Z: −8.51); TP9 (X: −23.30, Y: 70.08, Z: −42.09).

^3^
This measure was derived from an independent sample of 31 Italian native speakers (18 F, 13 M; mean age = 29.61 years, SD = 4.53 years) who rated the contextual availability on a 7‐point Likert scale (1 = very difficult to think about a context) of the word stimuli included in this study.

^4^
We defined such long epochs both to improve the following epoch‐based artifact rejection procedure and to prevent edge artifacts and obtaining a clean and stable baseline for time‐frequency decompositions, which we plan to perform and report in a future paper.

^5^
Note that the quadratic effects of Valence and Concreteness, which were included in the LMM model for the behavioral analyses, were not included in the LMM model for the EEG analysis. Indeed, we interpreted the quadratic effects on RTs as reflecting the difficulty of participants' decisions (e.g., it takes less time to decide whether a word is either positive or negative if its valence is either very high or very low, while it takes more time to perform the same affective decision task if the word has an intermediate (i.e., neutral) valence value), but we did not expect this quadratic effect to modulate participants' ERPs, as their voltage amplitude should be linearly modulated by valence/concreteness, reflecting the strength of their encoding and of the related cognitive processes.
