# Supplementary material for: Fine‐Grained Concreteness Effects on Word Processing and Representation Across Three Tasks: An ERP Study
Source: Psychophysiology. 2025 May 23;62(5):e70074. doi: 10.1111/psyp.70074 (PMC12100582; doi:10.1111/psyp.70074)
Supplement: Supplementary file 1 — Data S1. [file PSYP-62-e70074-s001.docx]

**Supplementary Information**

**Fine-Grained Concreteness Effects on Word Processing and Representation Across Three Tasks: An ERP Study**

Maria Montefinese^1,*^, Antonino Visalli^2,3^, Alessandro Angrilli^4,5^, Ettore Ambrosini^5,6^

^1^ Department of Developmental Psychology and Socialisation, University of Padova, Padova, Italy

^2^ IRCCS San Camillo Hospital, Venice, Italy

^3^ Department of Biomedical, Metabolic and Neuroscience, University of Modena and Reggio Emilia, Reggio Emilia, Italy

^4^ Department of General Psychology, University of Padova, Padova, Italy

^5^ Padova Neuroscience Center, University of Padova, Padova, Italy

^6^ Department of Neuroscience, University of Padova, Padova, Italy

***Correspondence:**

Maria Montefinese, Department of Developmental Psychology and Socialisation, University of Padova, Via Venezia 8, 35131 Padova, Italy. Email: maria.montefinese@unipd.it

**Supplementary Table 1 | Step 1 Backward reduced fixed-effect table**

|  | Eliminated | Sum Sq | NumDF | DenDF | F value | Pr(>F) |
| --- | --- | --- | --- | --- | --- | --- |
| Trial | 1 | 0.007 | 1 | 164.3 | 0.09 | .769 |
| Lett | 2 | 0.028 | 1 | 165.6 | 0.37 | .545 |
| OrtNB | 3 | 0.028 | 1 | 166.2 | 0.37 | .543 |
| WFr | 4 | 0.090 | 1 | 168.7 | 1.19 | .276 |
| WFr_OrtNB | 5 | 0.184 | 1 | 169.9 | 2.43 | .121 |
| PreRT | 0 | 9.854 | 1 | 9263.8 | 130.33 | <.001 |
| OLD20 | 0 | 0.348 | 1 | 169.6 | 4.60 | .033 |
| AoA | 0 | 1.971 | 1 | 169.2 | 26.06 | <.001 |

*Abbreviations*: Lett, number of letters; WFr, number of orthographic neighbours; WFr, word frequency; WFr_OrtNB, mean frequency of orthographic neighbours; PreRT, inverted response time at the preceding trial; OLD20, orthographic Levenshtein distance 20; AoA, age of acquisition; Sum Sq, sum of squares, NumDF, numerator degrees of freedom, DenDF, denominator degrees of freedom,

**Supplementary Table 2 | Step 2 Backward reduced fixed-effect table**

|  | Eliminated | Sum Sq | NumDF | DenDF | F value | Pr(>F) |
| --- | --- | --- | --- | --- | --- | --- |
| Val | 1 | <0.001 | 1 | 166.8 | <0.01 | .960 |
| Dom | 2 | 0.194 | 1 | 166.6 | 3.11 | .080 |
| PreRT | 0 | 4.484 | 1 | 9644.9 | 71.80 | <.001 |
| OLD20 | 0 | 0.293 | 1 | 167.6 | 4.70 | .032 |
| AoA | 0 | 1.260 | 1 | 168.1 | 20.18 | <.001 |
| Val^2 | 0 | 6.063 | 1 | 168.9 | 97.08 | <.001 |
| Aro | 0 | 0.262 | 1 | 168.1 | 4.20 | .042 |

*Abbreviations*: Val and Val^2, linear and quadratic valence; Dom, dominance; Aro, arousal. For other conventions see Supplementary Table 1.

**Supplementary Table 3 | Step 3 Backward reduced fixed-effect table**

|  | Eliminated | Sum Sq | NumDF | DenDF | F value | Pr(>F) |
| --- | --- | --- | --- | --- | --- | --- |
| ContAva | 1 | 0.004 | 1 | 165.6 | 0.05 | .818 |
| Ima | 2 | 0.012 | 1 | 163.8 | 0.18 | .676 |
| Val | 3 | 0.102 | 1 | 166.2 | 1.48 | .225 |
| Fam | 4 | 0.057 | 1 | 165.7 | 0.83 | .365 |
| Val^2 | 5 | 0.254 | 1 | 166.9 | 3.70 | .056 |
| Aro | 6 | 0.207 | 1 | 167.8 | 3.01 | .084 |
| PreRT | 0 | 1.167 | 1 | 9322.3 | 17.03 | <.001 |
| OLD20 | 0 | 0.526 | 1 | 168.5 | 7.67 | .006 |
| AoA | 0 | 0.296 | 1 | 168.9 | 4.31 | .039 |
| Con | 0 | 9.294 | 1 | 169.2 | 135.57 | <.001 |
| Con^2 | 0 | 3.396 | 1 | 168.8 | 49.54 | <.001 |

*Abbreviations*: ContAva, availability of contextual information; Ima, imageability; Fam, familiarity; Con and Con^2, linear and quadratic concreteness. For other conventions see Supplementary Tables 1 and 2.

**Supplementary Table 4 | Summary output of the final LMM model**

| **Predictors** | **Estimates** | **SE** | **t value** | **p** |
| --- | --- | --- | --- | --- |
| (Intercept) | -1.282 | 0.023 | -56.774 | <.001 |
| PreRT | 0.023 | 0.002 | 13.703 | <.001 |
| OLD20 | 0.019 | 0.006 | 3.304 | .001 |
| AoA | 0.036 | 0.007 | 5.460 | <.001 |
| Aro | 0.002 | 0.006 | 0.319 | .750 |
| Task [AFF] | 0.217 | 0.009 | 23.956 | <.001 |
| Task [SEM] | 0.212 | 0.009 | 23.257 | <.001 |
| Val | 0.003 | 0.006 | 0.511 | .609 |
| Val^2 | -0.023 | 0.010 | -2.274 | .023 |
| Con | -0.016 | 0.007 | -2.218 | .027 |
| Con^2 | -0.007 | 0.007 | -1.065 | .287 |
| Task [AFF]:Val | -0.016 | 0.004 | -3.961 | <.001 |
| Task [SEM]:Val | 0.011 | 0.004 | 2.793 | .005 |
| Task [AFF] :Val^2 | -0.068 | 0.006 | -11.380 | <.001 |
| Task [SEM]:Val^2 | 0.033 | 0.006 | 5.476 | <.001 |
| Task [AFF]:Con | 0.032 | 0.005 | 7.039 | <.001 |
| Task [SEM]:Con | -0.079 | 0.005 | -17.475 | <.001 |
| Task [AFF]:Con^2 | 0.019 | 0.005 | 4.302 | <.001 |
| Task [SEM]:Con^2 | -0.055 | 0.005 | -12.070 | <.001 |
| Marginal R2/Conditional R2: | 0.139 / 0.343 | | |  |

*Abbreviations*: Task[AFF], contrast between the grammatical and the affective decision task; Task[SEM], contrast between the grammatical and the semantic decision task; “:”, interaction; SE, standard error; R2, R-squared. For other conventions see Supplementary Tables 1-3.


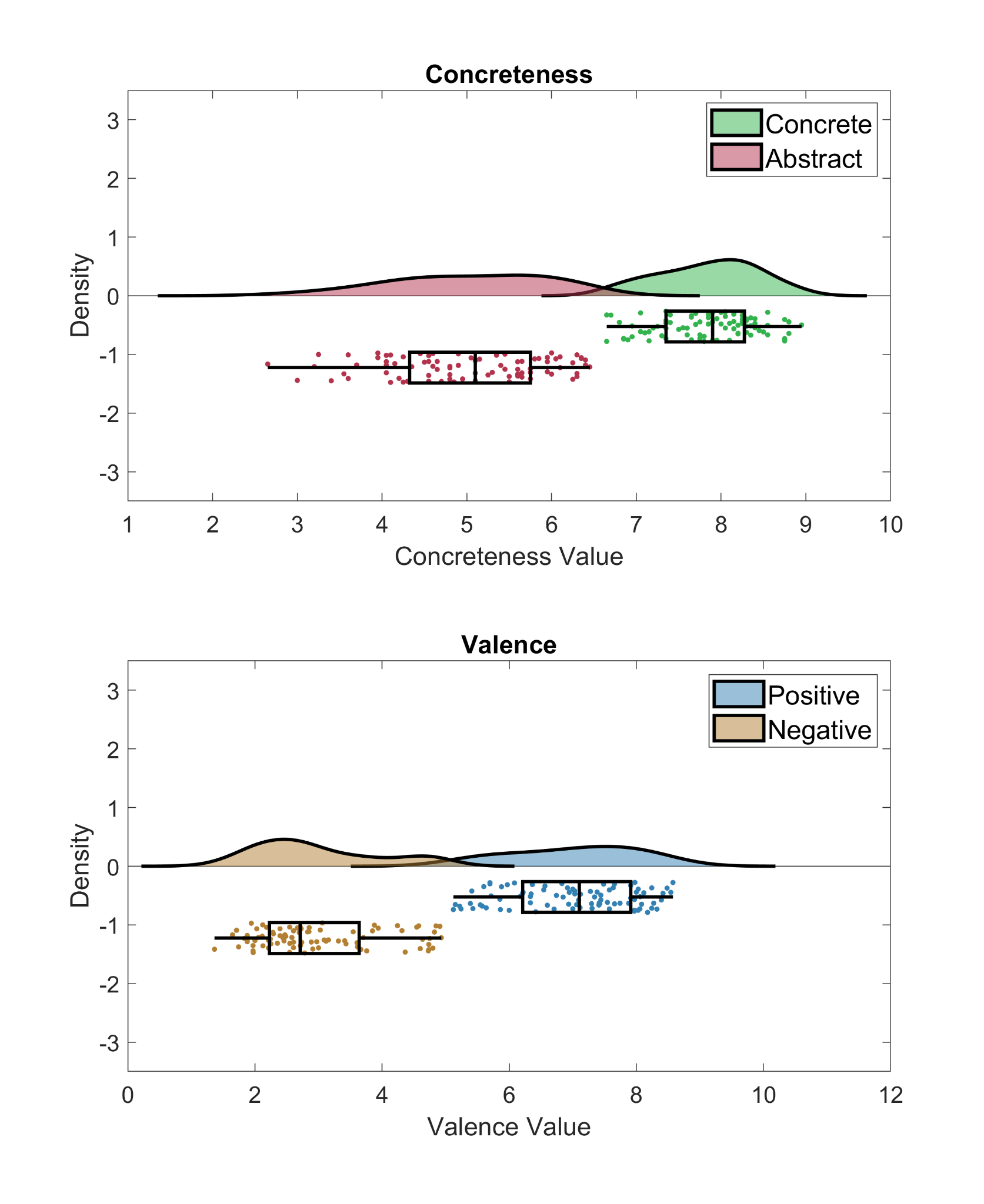


**Supplementary Figure 1 | Raincloud plots illustrating the distribution of concreteness and valence values for the stimuli used in the study.** (Upper panel) Distribution of concreteness values for concrete (green) and abstract (red) words, as identified by median split. (Lower panel) Distribution of valence values for positive (blue) and negative (orange) words, as identified by split.


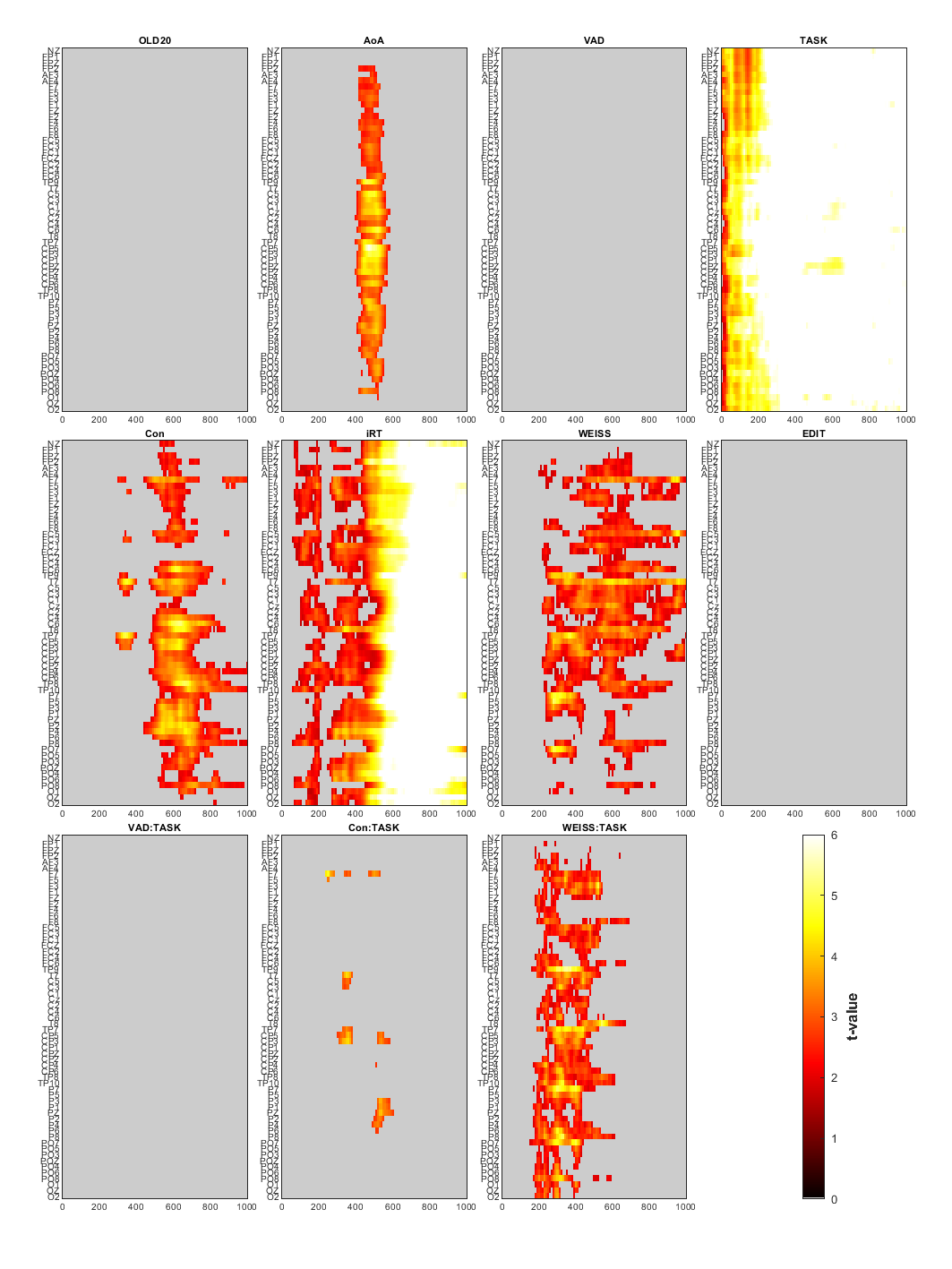


**Supplementary Figure 2 | RSA results (simple correlations).** The raster diagrams show electrodes/timepoints with significant EEG-model RSs for the model indicated in the corresponding heading. Grey rectangles indicate electrodes/time points for which no significant EEG-model RSs were observed. See Figure 5 for conventions.


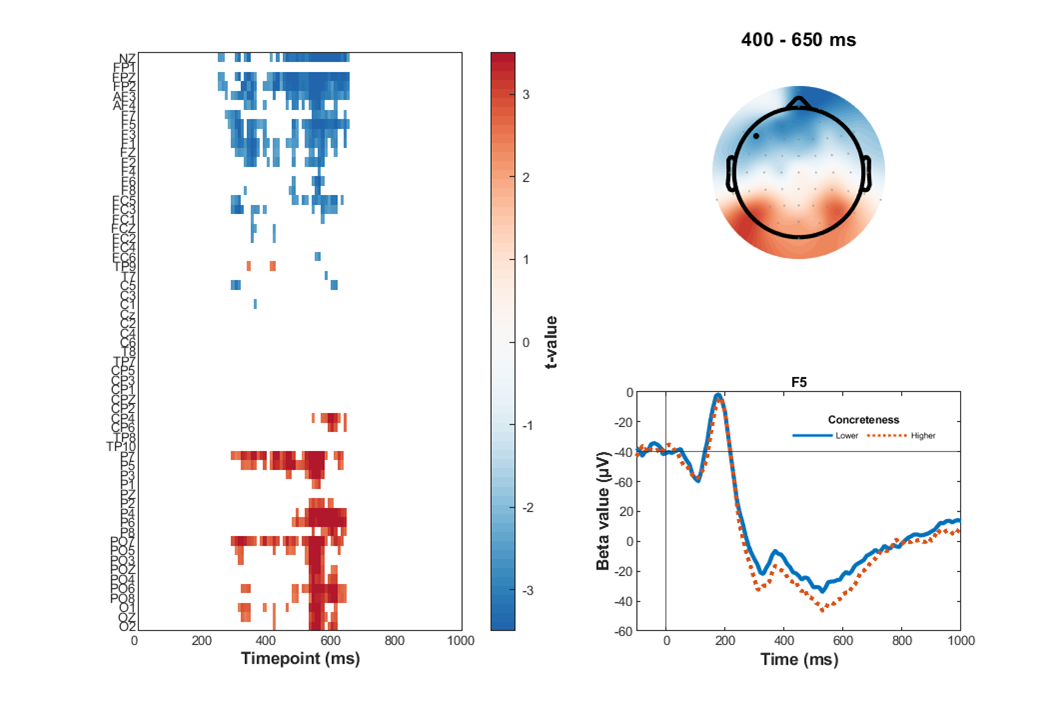


**Supplementary Figure 3 | Significant effects elicited by the Concreteness in a lmeEEG model including only Concreteness × Task interactions and lower simple effects (no confounding variables).** The raster diagram (left) shows electrodes/timepoints significantly modulated by Concreteness. White rectangles indicate electrodes/time points for which no significant modulations were observed. The topoplot shows the t values (same colour scale as the raster diagram) averaged in the indicated time windows. The trace-plot depicts the LM estimated responses for higher (orange dotted line) and lower (blue solid line) concreteness in the grammatical task for the electrode F5.
